# A Redox Regulatory System Critical for Mycobacterial Survival in Macrophages and Biofilm Development

**DOI:** 10.1371/journal.ppat.1004839

**Published:** 2015-04-17

**Authors:** Kerstin A. Wolff, Andres H. de la Peña, Hoa T. Nguyen, Thanh H. Pham, L. Mario Amzel, Sandra B. Gabelli, Liem Nguyen

**Affiliations:** 1 Department of Molecular Biology and Microbiology, School of Medicine, Case Western Reserve University, Cleveland, Ohio, United States of America; 2 Department of Biomedical Engineering, School of Medicine, Johns Hopkins University, Baltimore, Maryland, United States of America; 3 Department of Biophysics and Biophysical Chemistry, School of Medicine, Johns Hopkins University, Baltimore, Maryland, United States of America; 4 Department of Medicine, School of Medicine, Johns Hopkins University, Baltimore, Maryland, United States of America; 5 Department of Oncology, School of Medicine, Johns Hopkins University, Baltimore, Maryland, United States of America; National Institutes of Health, UNITED STATES

## Abstract

Survival of *M*. *tuberculosis* in host macrophages requires the eukaryotic-type protein kinase G, PknG, but the underlying mechanism has remained unknown. Here, we show that PknG is an integral component of a novel *r*edox *ho*meostati*c* *s*ystem, RHOCS, which includes the ribosomal protein L13 and RenU, a Nudix hydrolase encoded by a gene adjacent to *pknG*. Studies in *M*. *smegmatis* showed that PknG expression is uniquely induced by NADH, which plays a key role in metabolism and redox homeostasis. *In vitro*, RenU hydrolyses FAD, ADP-ribose and NADH, but not NAD+. Absence of RHOCS activities *in vivo* causes NADH and FAD accumulation, and increased susceptibility to oxidative stress. We show that PknG phosphorylates L13 and promotes its cytoplasmic association with RenU, and the phosphorylated L13 accelerates the RenU-catalyzed NADH hydrolysis. Importantly, interruption of RHOCS leads to impaired mycobacterial biofilms and reduced survival of *M*. *tuberculosis* in macrophages. Thus, RHOCS represents a checkpoint in the developmental program required for mycobacterial growth in these environments.

## Author Summary

Nearly one-third of the world’s population is infected with *Mycobacterium tuberculosis* (*Mtb*), the causative agent of TB. A key factor that contributes to the widespread infection of *Mtb* is its capacity to survive inside the host macrophage. Understanding how *Mtb* withstands the hostile intracellular environment of this phagocytic cell may reveal targets for development of therapeutics that enhance the innate anti-*Mtb* activities of the macrophage. We discovered a novel signaling pathway in mycobacteria which regulates cellular redox homeostasis through NADH and FAD, regulators of metabolism and redox balance. NADH induces the expression of a protein kinase, PknG, which then phosphorylates the ribosomal protein L13 and promotes its presence in the cytoplasm. L13 therein forms a complex with RenU, a Nudix (*Nu*cleoside *di*phosphate linked moiety X) hydrolase that degrades NADH and FAD. Genetic disruption of this signaling cascade leads to cellular accumulation of these molecules, increased mycobacterial sensitivity to oxidative stress, impaired surface biofilm growth, and most importantly, reduced survival of *Mtb* in macrophages.

## Introduction

A critical determinant defining pathogenicity of *Mycobacterium tuberculosis* (*Mtb*) is its survival in host macrophages. Upon internalization by the host phagocytic cell, *Mtb* and related pathogenic mycobacteria block the fusion of their resident phagosome to the destructive lysosome, thereby establishing a niche within the bactericidal macrophage [1,2]. This ability of pathogenic mycobacteria requires the eukaryotic-type serine/threonine protein kinase G (PknG) [3]. Lack of PknG activity results in rapid delivery of mycobacteria to lysosomes, leading to enhanced killing of the intracellular bacilli by macrophages [3]. Besides its role in the innate survival of *Mtb* in host cells, PknG provides mycobacterial species with an intrinsic resistance to antibiotics [4]. In the absence of PknG, both pathogenic *Mtb* and non-pathogenic *M*. *smegmatis* display increased susceptibility to multiple antibiotics [4]. These observations suggest that PknG might be required for the persistence of *Mtb* within hosts, during which the bacillus also becomes highly recalcitrant to antibiotics. Although PknG represents an attractive target for tuberculosis (TB) drug development, the molecular mechanism by which this kinase exerts its biological functions remains largely unknown. It was shown that PknG is secreted via the SecA2 secretion system [5] into the macrophages’ cytosol [3] where it is hypothesized to interfere with host signaling pathways controlling phagolysosome synthesis [3]. However, attempts to identify the putative host substrate(s) targeted by PknG have thus far been unsuccessful. As a result, the role of PknG in *Mtb* survival in host macrophages remains ambiguous.

In other pathogenic bacteria such as *Vibrio cholerae*, *Escherichia coli*, *Pseudomonas aeruginosa*, *Streptococcus* sp., and *Haemophilus influenza*, host persistence and antibiotic tolerance are tightly correlated with the ability to form biofilms, community-like growth consisting of surface-bound cells that are metabolically and physiologically distinct from planktonic cells [6–9]. *In vitro*, *Mtb* and other mycobacterial species can form biofilms, which require iron and mobile mycolate moieties [10–12]. Development of mycobacterial biofilms is also modulated by activities of enzymes of the tricarboxylic acid (TCA) cycle, such as 2-oxoglutarate dehydrogenase [11]. Like other bacteria, mycobacterial cells persisting within biofilms display increased antibiotic tolerance, reminiscent of *Mtb* cells that form during latent TB [11]. However, it has remained largely unknown how the antibiotic-tolerant biofilm of *Mtb* relates to its pathogenicity, and whether these phenotypic correlations are co-regulated in *Mtb* and related mycobacteria.

Here, we found that in mycobacteria, biofilm growth and host persistence are both regulated by RHOCS, a newly identified redox homeostatic system in which PknG plays a central role. We show that the redox regulatory molecule NADH induces the expression of PknG, which phosphorylates the ribosomal protein L13 at a unique residue, threonine 11 (T11). The phosphorylation promotes the cytoplasmic association of L13 with RenU, a Nudix hydrolase encoded by a gene adjacent to *pknG* on mycobacterial chromosomes, and accelerates RenU’s NADH hydrolytic activity. Disruption of the PknG-L13-RenU pathway causes: (i) increased oxidative stress susceptibility, (ii) accumulation of NADH and FAD during oxidative stress, (iii) impaired biofilm growth, and notably, (iv) reduced survival of *Mtb* in host macrophages. These results suggest that biofilm growth and host persistence are both regulated through the PknG-modulated RHOCS, which regulates levels of nucleoside diphosphate derivatives such as NADH and FAD in mycobacteria.

## Results

### Kinase Activity of PknG Is Required for Biofilm Growth in Mycobacteria

**Fig 1:**
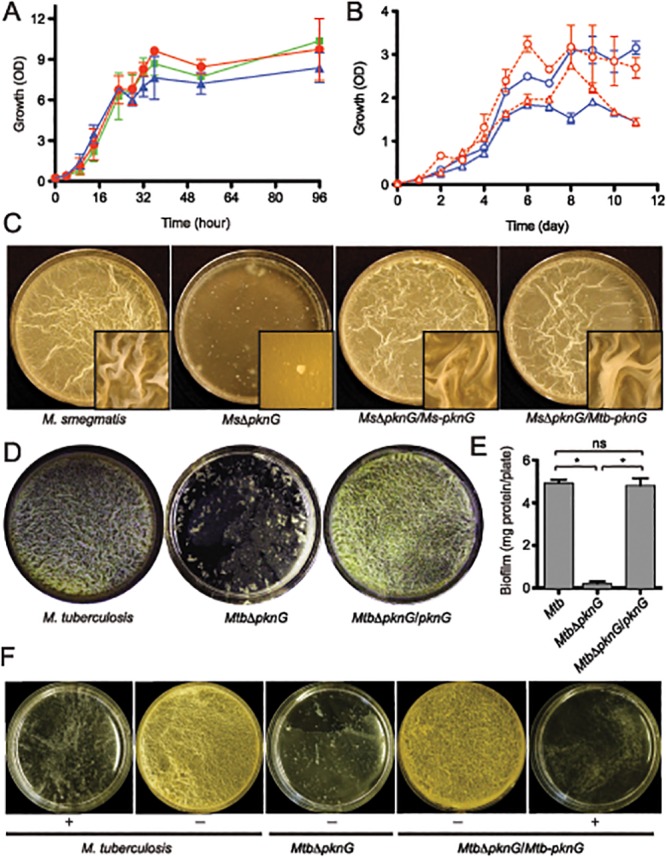
PknG kinase activity is required for biofilm growth in mycobacteria. (**A**) Role of *pknG* in *M*. *smegmatis* planktonic growth. Wild type *M*. *smegmatis* mc^2^155 (red filled circles), its derived *Ms*Δ*pknG* mutant (blue filled triangles), and the complemented strain *Ms*Δ*pknG*/*pknG* (green filled squares) were grown in 7H9 medium supplemented with 0.2% glucose with shaking at 200 r.p.m. and 37°C. Growth was assessed by measuring optical absorbance at 600 nm. Error bars represent standard deviation of biological triplicates. Differences between wild type and *Ms*Δ*pknG* in stationary phase are not significant. (**B**) Role of *pknG* in *Mtb* planktonic growth. Wild type *Mtb* H37Rv (open circles) and its derived *Mtb*Δ*pknG* mutant (open triangles) were grown in 7H9-OADC medium with 0.2% glucose (blue) or 1% glucose (red). Cultures were shaken at 200 r.p.m. and 37°C. Growth was assessed by measuring optical absorbance at 600 nm. Error bars represent standard deviation of biological triplicates. Differences between wild type and *Mtb*Δ*pknG* in stationary phase (5–11 hours) are statistically significant (two-tailed *t*-test, p<0.05). (**C**) *pknG* is required for *M*. *smegmatis* biofilm growth. Wild type *M*. *smegmatis*, *Ms*Δ*pknG*, and the mutant strains complemented with the *M*. *smegmatis* (*Ms-pknG*) or *M*. *tuberculosis* (*Mtb-pknG*) gene. Pictures were taken after 7 days of static growth at 30°C. Shown images are representatives of biological triplicates. (**D**) *pknG* is required for *Mtb* biofilm growth. Wild type *Mtb* H37Rv, *Mtb*Δ*pknG*, and the complemented strain were assayed as previously described [10]. Pictures were taken after 6 weeks of growth at a static humidified condition of 37°C and 5% CO_2_. Shown images are representatives of biological triplicates. (**E**) Quantitation of biofilm growth of *Mtb* strains. Biofilms were harvested and quantified as described in Experimental Procedures. Error bars represent standard deviation of biological triplicates (*, p<0.0001; ns, not significant difference between wild type H37Rv and the complemented strain). (**F**) PknG kinase activity is required for *Mtb* biofilm growth. Wild type *Mtb* H37Rv, *Mtb*Δ*pknG*, and the complemented strain were assayed in the absence (-) or presence (+) of 1 mM AX20017, a specific inhibitor of PknG. Pictures were taken after 6 weeks of growth at static humidified condition of 37°C and 5% CO_2_. Shown images are representatives of biological triplicates.

Studies in other bacteria have established the phenotypic relationship between host persistence, antibiotic resistance and biofilm growth [6,9]. PknG was previously shown to be required for two of these three phenotypes, namely survival in macrophages and resistance to multiple antibiotics [3,4]. In addition, phenotypic characterization of a *M*. *smegmatis* Δ*pknG* mutant revealed profound alterations in surface charge and hydrophobicity of the cell wall (S1 Table) [4], suggesting that PknG might be involved in mycobacterial biofilm growth. Wild type strains of *M*. *smegmatis*, *M*. *bovis* BCG, and *Mtb*, together with their derived Δ*pknG* mutants and complemented strains were assayed for growth in both planktonic cultures and static biofilms (Figs 1 and S1). Similar to *M*. *bovis* BCG [3,13], absence of PknG did not affect planktonic growth of *M*. *smegmatis* (Fig 1A). However, the *Mtb*Δ*pknG* displayed a slow growth defect in stationary phase (Fig 1B), as previously reported [14]. These phenotypic variations suggest a complexity of PknG function among mycobacterial species that warrants further investigation.

However, in all mycobacterial species investigated, Δ*pknG* mutants consistently displayed severe retardation in biofilm growth (Figs 1C–1F and S1). As previously described [10], wild type cells initially formed clusters emerging onto the surface, which then steadily spread and eventually covered the entire liquid-air interface. This surface invasion was followed by a maturation stage characterized by the formation of typical surface wrinkles (Figs 1C–1F and S1). By contrast, clusters of Δ*pknG* cells formed unevenly and failed to cover the entire surface. Many of these cell clusters eventually sank and became submerged in the liquid phase (Figs 1C–1F and S1). A surface attachment assay also showed insufficient surface dispersal by *M*. *smegmatis* Δ*pknG* (S2 Fig). Biofilm growth of the Δ*pknG* mutants was completely restored by *in trans* expression of either intraspecific or interspecific *pknG* genes (Fig 1C–1F), suggesting that PknG provides similar functions in biofilm growth to all mycobacterial species.

To test if the requirement for PknG is due to its kinase activity, biofilm growth of *Mtb* strains was assayed in the presence or absence of a PknG-specific inhibitor, AX20017 [3,15]. Similar to genetic deletions (Figs 1C–1E and S1), AX20017 completely abolished biofilm growth of both wild type and the complemented strain of *Mtb* (Fig 1F), whereas it had no effect on planktonic growth in similar growth media [3,15]. Collectively, these results show that PknG kinase activity is required for growth of mycobacteria including *Mtb* in the static condition of surface biofilms.

### Both *pknG* and Its Neighboring Nudix Hydrolase Gene *renU* Are Involved in Redox Homeostasis

**Fig 2:**
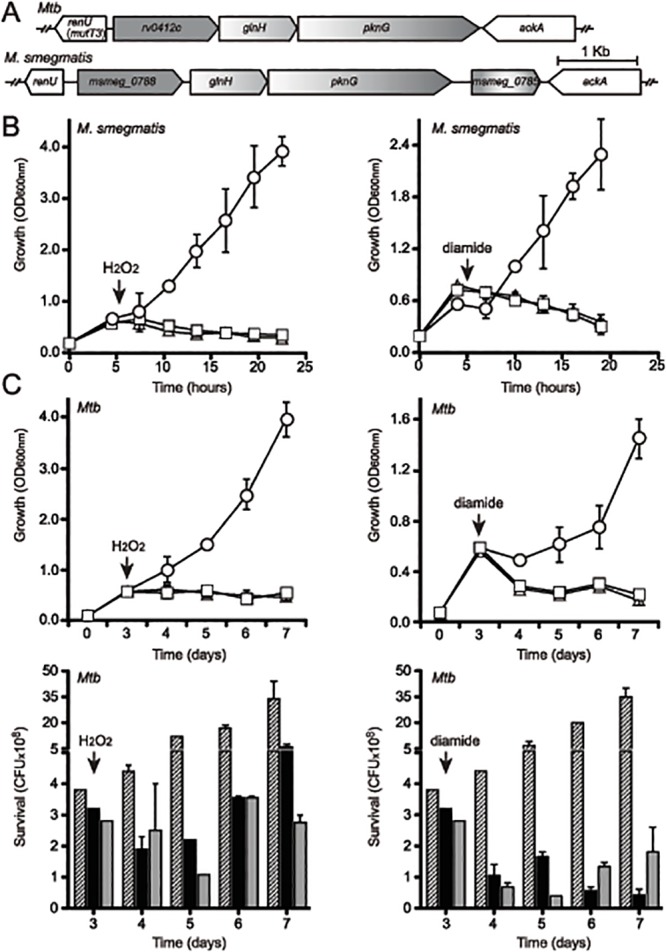
Both *pknG* and its adjacent gene *renU* are each required for oxidative stress resistance. (**A**) Alignment of the *pknG* loci from *Mtb* and *M*. *smegmatis*. *renU* (previously annotated as *mutT3*) shares the same intergenic region with the operon encoding *pknG*. Bar, 1kb. (**B**) Both *pknG* and *renU* are each required for *M*. *smegmatis* resistance to H_2_O_2_ (left) and diamide (right). Wild type *M*. *smegmatis* (circles), *Ms*Δ*pknG* (triangles) and *Ms*Δ*renU* (squares) were grown in 7H9 medium. At the indicated times (arrows), 10mM H_2_O_2_ or 15mM diamide was added. Growth was estimated through optical absorbance at 600 nm (OD_600nm_). Error bars represent standard deviation of biological triplicates. (**C**) *pknG* and *renU* are each required for *Mtb* resistance to H_2_O_2_ (left) and diamide (right). Wild type *Mtb* (circles or striped bars), *Mtb*Δ*pknG* (triangles or black filled bars) and *Mtb*Δ*renU* (squares or grey filled bars) of were grown in 7H9-OADC medium. At the indicated times (arrows), 20 mM H_2_O_2_ or 10 mM diamide was added. Growth was estimated through measuring optical absorbance at 600 nm (OD_600nm_, top) or determining colony forming units (CFU, bottom) by serial dilution plating. Error bars represent standard deviation of biological triplicates.

Structural studies revealed a rubredoxin-like domain at the N-terminus of PknG, suggesting a possible involvement of this kinase in redox homeostasis [15]. In fact, our *in vitro* phosphorylation assays supported the hypothesis that PknG kinase activity may be regulated by the redox state of the mycobacterial cytoplasm (S3 Fig). We studied the role of the *pknG* locus in mycobacterial redox homeostasis. Interestingly, these studies revealed that *pknG* and a neighboring gene (*msmeg_0790*/*rv0413*), previously annotated as *mutT3* (Fig 2A), were each required for oxidative stress resistance in *M*. *smegmatis* and *Mtb* (Fig 2B–2C). The deduced amino acid sequences encoded by *msmeg_0790* and *rv0413* show the motif GX_5_EX_7_REUXEEXGU (where U = L,V,I), typical of Nudix (*Nu*cleoside *di*phosphate linked moiety *X*) hydrolases [16]. Nudix hydrolases are low molecular weight (MW) “housecleaning” phosphohydrolases that provide control over cellular levels of deleterious metabolic intermediates [16]. The proteins encoded by *msmeg_0790* and *rv0413* had been wrongly annotated as MutT3 because they were thought to be anti-mutators, prototypical Nudix hydrolases that degrade and prevent misincorporation of 8-oxo-guanosine triphosphate into nucleic acids. However, recent studies showed that *msmeg_0790* and *rv0413* are not involved in anti-mutation activity [17].

Wild type *M*. *smegmatis* and *Mtb*, their derived *Ms*Δ*pknG* and *Mtb*Δ*pknG* mutants, and Δ*msmeg_0790* or Δ*rv0413* mutants, respectively, were challenged with oxidative stress triggered by H_2_O_2_ (Fig 2B–2C, left panels) or diamide (Fig 2B–2C, right panels). In both *M*. *smegmatis* (Fig 2B) and *Mtb* (Fig 2C) backgrounds, addition of H_2_O_2_ or diamide completely stopped the growth of the mutants, whereas the wild type strains continued to grow. These results suggest that both *pknG* and *msmeg_0790*/*rv0413* are involved in a redox regulatory mechanism that protects mycobacteria from oxidative stress. In light of the fact that this protein does not function as an anti-mutator, and the findings described in this paper, we propose to rename this Nudix hydrolase as RenU (for *Re*dox *Nu*dix hydrolase).

### Nudix Hydrolase Activity of RenU and Its Requirement for Biofilm Growth

**Fig 3:**
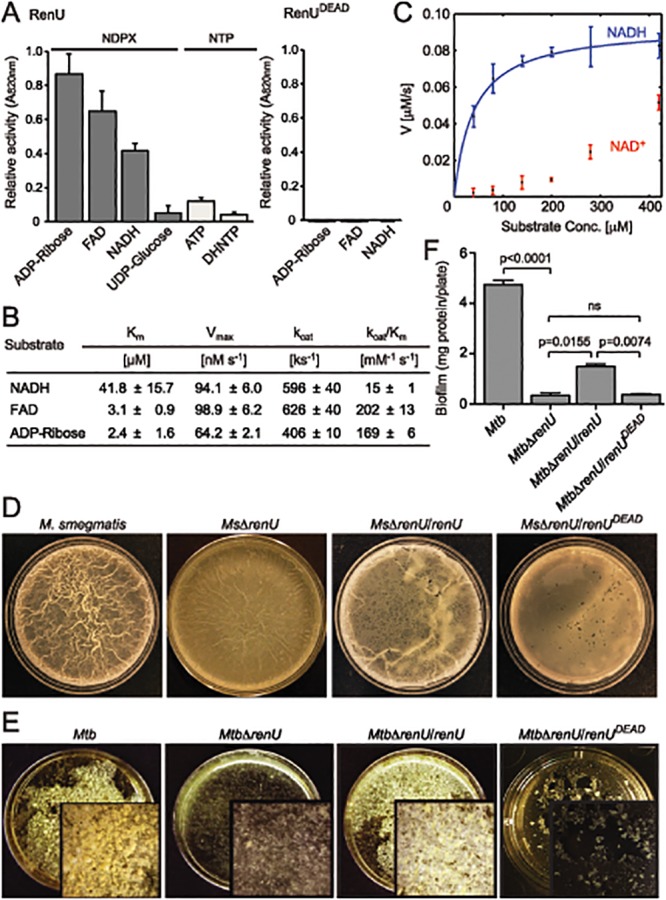
*renU* encodes a Nudix hydrolase required for biofilm growth. (**A**) Relative Nudix hydrolase activity of RenU on a substrate panel (left). Nucleoside diphosphate derivatives (NDPX) are preferred substrates compared to nucleoside triphosphates (NTP). A catalytically-inactive mutant of RenU (RenU^DEAD^) protein, in which 3 glutamate residues (E74, E77, and E78) in the Nudix box were mutated to alanines, exhibits no phosphatase activity towards the preferred substrates (right). (**B**) Kinetics studies of Nudix hydrolase activity of RenU on the three NDPXs as preferred substrates ADP-ribose, FAD, and NADH. (**C**) Rate of RenU catalytic activity on NADH compared to its oxidative form NAD^+^. Fit curve is shown for NADH. (**D**) The Nudix hydrolase activity of RenU is required for *M*. *smegmatis* biofilm growth. Wild type *M*. *smegmatis*, *Ms*Δ*renU*, and the mutant strains completed with wild type RenU or RenU^DEAD^ were assayed for biofilm growth. Whereas *renU* fully restored biofilm growth to *Ms*Δ*renU*, *renUDEAD* failed to complement the mutant. Shown images are representatives of biological triplicates. (**E**) The Nudix hydrolase activity of RenU is required for *Mtb* biofilm growth. Wild type *Mtb* H37Rv, *Mtb*Δ*renU*, and the mutant strains completed with wild type RenU or RenU^DEAD^ were was assayed for biofilm growth. Whereas *renU* fully restored biofilm growth to *Mtb*Δ*renU*, *renUDEAD* failed to complement the mutant. Shown images are representatives of biological duplicates. (**F**) Quantitation of biofilm growth of *Mtb* strains. The biofilm biomass was harvested and estimated by determining total protein per plate. Error bars represent standard deviation of biological triplicates. Statistical significances of differences were analyzed using Students *t*-test; ns, not significant difference.

To test whether RenU is indeed a hydrolase, and to further characterize its enzymatic activity, the recombinant *M*. *smegmatis* RenU was purified to homogeneity. Size exclusion chromatographic analysis showed that RenU was monomeric in solution (S4 Fig). Next, its enzymatic activity was determined using a coupled enzyme colorimetric assay described in the Extended Experimental Procedures. Substrate specificity was investigated with a panel of several different nucleoside diphosphate derivatives (NDPX) and nucleoside triphosphates (NTP). RenU did exhibit Nudix hydrolase activity with a substrate preference for NDPXs. Among the substrates tested, the highest activities were observed with ADP-ribose, FAD, and NADH (Figs 3A left panel, and S5). By contrast, the enzyme displayed much lower activities towards NTPs including ATP, 7,8-dihydroneopterin triphosphate (DHNTP) (Fig 3A left panel), dGTP, dCTP, dUTP, or other NDPXs such as CoA, GDP-D-mannose, NADP, ADP-ADP, and CDP-choline (S5 Fig). Importantly, mutations in glutamate residues of the Nudix box (E74, E77, and E78, see S2 Table), which are expected to coordinate the magnesium required for the activities of Nudix hydrolases, completely abolished the enzymatic activity of RenU. The mutated protein, RenU^DEAD^, displayed no activity towards the preferred substrates exhibited by wild type RenU (Fig 3A, right panel). Michaelis-Menten analysis revealed that, *in vitro*, ADP-ribose and FAD were better substrates than NADH, as evidenced by its higher k_cat_/K_m_ value (Figs 3B and S6). However, previous studies with Nudix hydrolases predict that the substrate preference of RenU might be defined *in vivo* through its interactions with other proteins [18]. In fact, analysis of cellular levels and PknG induction experiments (see below) suggest that NADH is the physiologically relevant substrate of RenU *in vivo*. Interestingly, while RenU readily hydrolyzed NADH, the reduced form of nicotinamide adenine dinucleotide, it did not show significant catalytic activity towards the oxidized form, NAD^+^ (Fig 3C).

To determine whether the Nudix hydrolase activity of RenU is required for mycobacterial biofilm growth, *Ms*Δ*renU* and *Mtb*Δ*renU* mutants, their parental wild types, and the mutants complemented with either RenU or RenU^DEAD^, were tested in biofilm growth assays. As shown in Fig 3D–3F, the Δ*renU* mutants were as defective as the Δ*pknG* mutants in biofilm growth. Whereas *in trans* expression of RenU partially restored biofilm growth, expression of the RenU^DEAD^ form failed to rescue the biofilm in both the Δ*renU* mutants (Fig 3E–3F), confirming the requirement for this Nudix hydrolase activity in mycobacterial biofilm growth. These observations, together with their cognate chromosomal localization, further suggest that PknG and RenU participate in the same pathway required for mycobacterial biofilm growth.

### RenU Forms a Complex with Ribosomal Protein L13, A Novel Substrate of PknG

**Fig 4:**
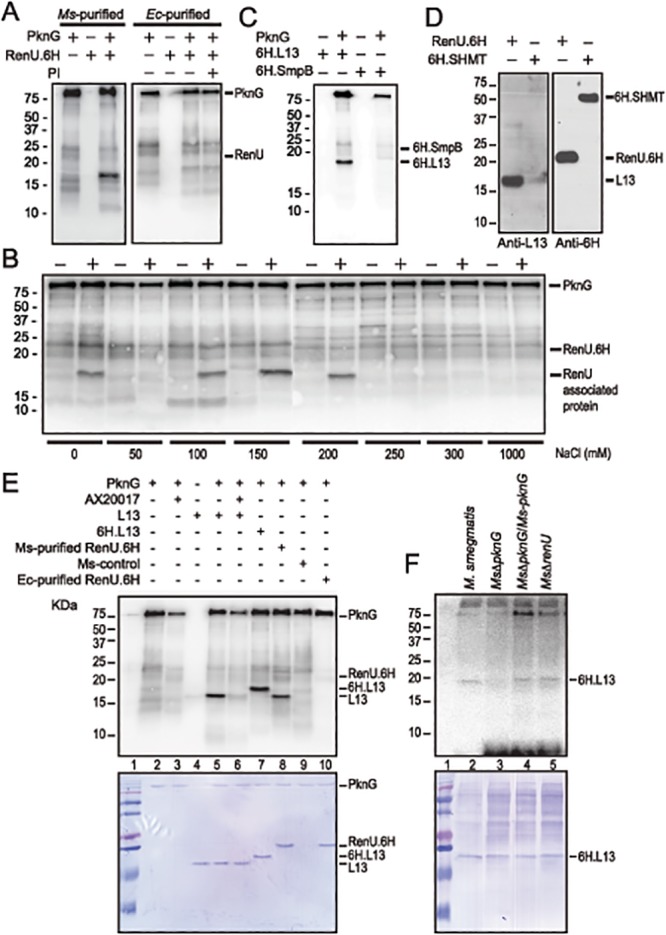
L13, a ribosomal protein associated with RenU, is phosphorylated by PknG. (**A**) Representative *in vitro* phosphorylation of RenU.6H preparations purified from *M*. *smegmatis* (left) or *E*. *coli* (right) by purified PknG. PI, phosphatase inhibitors. (**B**) *In vitro* phosphorylation of corresponding fractions eluted from ion exchange columns by PknG. Numbers indicate the NaCl concentrations used in elution buffer. Samples loaded to the ion exchange columns were obtained from an immobilized Cobalt affinity chromatography of *M*. *smegmatis* RenU.6H (+) cell lysates or control lysates (-). (**C**) *In vitro* phosphorylation of purified 6H.L13 or 6H.SmpB by PknG. (**D**) Co-purification of L13 from *M*. *smegmatis* lysates by exogenous RenU.6H. Another recombinant 6H-tagged protein (6H.SHMT) was used as a control. Blots were detected by Anti-L13 or Anti-6H antibodies. (**E**) *In vitro* phosphorylation of recombinant or native L13 protein associated with RenU by PknG kinase activity. (**F**) *In vitro* phosphorylation of purified 6H.L13 by *M*. *smegmatis* cell lysates, followed by pull-down using Nickel-agarose beads.

To investigate how PknG and RenU interact, we first tested if PknG phosphorylates RenU. The encoding genes were cloned for expression in *M*. *smegmatis* or *E*. *coli* as 6xHistidine-(6H) tagged proteins. Purified RenU.6H preparations were subjected to *in vitro* kinase assays using radioactive [γ-P^32^]-ATP as the phosphate donor. Whereas the *M*. *smegmatis*-derived RenU.6H displayed a protein species phosphorylated by PknG, the *E*. *coli*-derived equivalent did not show phosphorylation (Fig 4A). This was unlikely due to contaminated phosphatases because addition of phosphatase inhibitors (PI) did not reverse the phosphorylation pattern (Fig 4A).

The MW of the phosphorylated protein species found in the *M*. *smegmatis*-purified RenU.6H appeared ~5 kDa smaller than RenU.6H, as revealed by Coomassie Blue stained gels (Fig 4A and 4E, lane 8). In addition, corresponding fractions obtained during the purification of a control cell lysate (from a *M*. *smegmatis* strain carrying only the expression plasmid without the RenU-encoding sequence) did not show the same phosphorylated protein species (Fig 4B). These results suggest that the phosphorylated protein was of *M*. *smegmatis* origin and associated with RenU.

To identify the RenU-associated protein that is phosphorylated by PknG, the identities of proteins co-purified with RenU.6H expressed in *M*. *smegmatis* were analyzed by mass spectrometry. Among the 15 proteins found to associate with RenU (S3 Table), three had theoretical MWs similar to the phosphorylated protein: *ssrA*-binding protein SmpB (MW 18,287), 50S ribosomal protein L13 (or RplM, MW 16,119), and 50S ribosomal protein L16 (or RplP, MW 15,595). *M*. *smegmatis* genes encoding these proteins were cloned and expressed in *E*. *coli* as 6H-tagged proteins. The recombinant proteins were purified and subjected to *in vitro* phosphorylation assays. These experiments showed that only L13 was readily phosphorylated by PknG (Fig 4C and 4E, lane 5). Co-purification experiments confirmed the interaction of L13 and RenU in the cytoplasm of *M*. *smegmatis* (Fig 4D). In addition, the MW displayed by L13 on Coomassie Blue gels and autoradiographs was identical to the protein previously found to associate with RenU purified from *M*. *smegmatis* (Fig 4E, lanes 5 and 8). Without PknG, L13 proteins from either *M*. *smegmatis* or *Mtb* showed no sign of phosphorylation in the presence of [γ-P^32^]-ATP (Fig 4C and 4E, lanes 4), showing that the phosphorylation requires PknG. Furthermore, addition of the PknG specific inhibitor AX20017 inhibited the phosphorylation of L13 by PknG (Fig 4E, lane 6). Addition of a 6H-tag shifted the phosphorylated signal of L13 visualized on autoradiograph and Coomassie Blue stained gel (Fig 4E, lane 7). These results confirmed the phosphorylation of L13 by the kinase activity of PknG. Similar to its autophosphorylation (S3 Fig, panel A), the *in vitro* phosphorylation of L13 by PknG is affected by the redox status of the environment (S3 Fig, panel B).

To further establish whether L13 phosphorylation is PknG specific, *in vitro* phosphorylation assays were carried out using 6H.L13 as substrate, and cell lysates from *M*. *smegmatis* strains as the kinase sources. Using [γ-P^32^]-ATP as the phosphate donor, the *in vitro* phosphorylation assays were followed by pull-down of 6H.L13 using nickel-NTA-agarose beads (Qiagen). Precipitated materials were washed, treated with SDS sample buffer and released proteins separated on SDS-PAGE gels, followed by autoradiography. Whereas lysates from *M*. *smegmatis* strains expressing PknG, either in trans or chromosomally, readily phosphorylated 6H.L13, lysates from *Ms*Δ*pknG* failed to do so, indicating that phosphorylation of L13 specifically requires PknG (Fig 4F). It is also important to note that the phosphorylation of L13 by PknG did not require RenU (Fig 4F, lane 5), suggesting that phosphorylation occurs before L13 and RenU form a complex.

### Phosphorylation Status of L13 Affects Biofilm Growth

**Fig 5:**
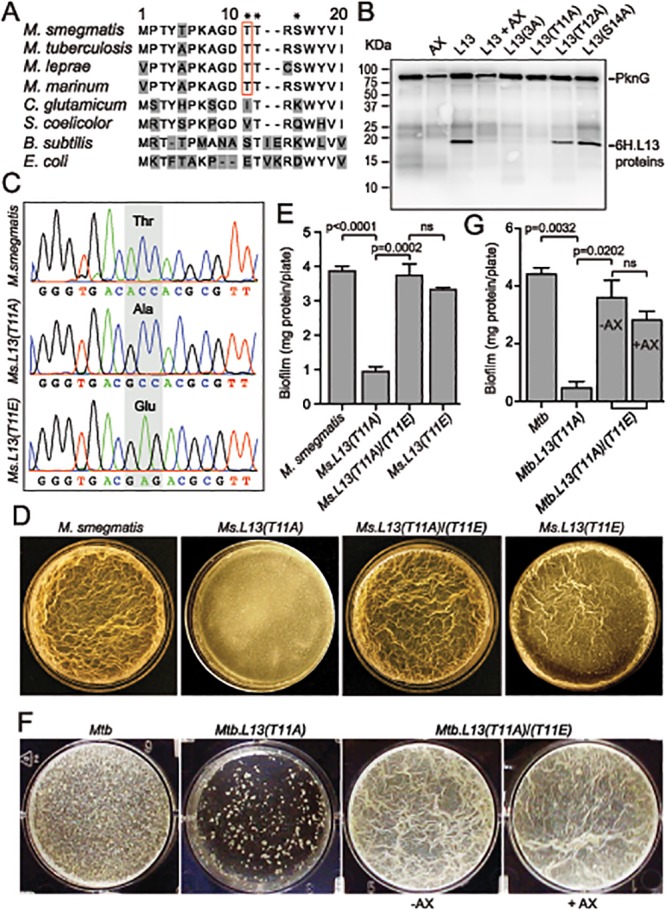
PknG-catalyzed phosphorylation of L13 at a mycobacterial specific site, T11, is required for mycobacterial biofilm growth. (**A**) Sequence alignment of the N-terminal 20 amino acids of L13 proteins from different bacteria. Residues marked with asterisks (T11, T12, and S14) are potential targets of phosphorylation by PknG. (**B**) Phosphorylation of L13 and its mutants by PknG. In L13(3A), all three residues (T11, T12, and S14) were mutated to alanine. Inhibition was achieved by pre-incubation of PknG in 1 mM AX20017 (AX). (**C**) Chromatograms confirming wild type and mutant alleles of *rplM* on *M*. *smegmatis* chromosomes. The chromosomal loci were amplified from genomic DNA of *M*. *smegmatis* strains by primers that anneal to DNA sequences outside the regions homologous to the allelic exchange substrates, followed by cloning and sequencing. (**D**) Biofilm of *M*. *smegmatis* strains. Similar to *Ms*Δ*pknG* and *Ms*Δ*renU*, *Ms*.*L13(T11A)* exhibited defective biofilm growth, while biofilm of *Ms*.*L13(T11E)* was largely identical to wild type. *In trans* expression of an allele encoding L13(T11E) restored biofilm growth to *Ms*.*L13(T11A)* strain. (**E**) Quantitation of biofilm growth of *M*. *smegmatis* strains. The biofilm biomass was harvested and quantified by determining total protein per plate. Error bars represent standard deviations of biological triplicates. Statistical significances of differences were analyzed using Students *t*-test; ns, not significant difference. (**F**) Biofilm of *Mtb* strains. Similar to *Mtb*Δ*pknG* and *Mtb*Δ*renU*, *Mtb*.*L13(T11A)* exhibited defective biofilm growth while *in trans* expression of an allele encoding L13(T11E) restored its biofilm growth. Addition of PknG inhibitor AX20017 (+AX) had no effect on the biofilm of the complemented strain. (**G**) Quantitation of biofilm growth of *Mtb* strains. The biofilm biomass was harvested and quantified by determining total protein per plate. Error bars represent standard deviations of biological triplicates. Statistical significances of differences were analyzed using Students *t*-test; ns, not significant difference.

L13 proteins, encoded by *rplM* genes from both *M*. *smegmatis* (Fig 4C and 4E) and *Mtb* (Fig 5), were equally phosphorylated by PknG, indicating that this phosphate transfer reaction serves an identical function in mycobacteria. To identify the specific amino acid residues that are phosphorylated by PknG, *Mtb* L13 protein purified from *E*. *coli* was subjected to a cold kinase assay catalyzed by PknG. L13 was then digested with trypsin and the derived peptides were analyzed by ISL-TOFF mass spectrometry (Taplin Biological Mass Spectrometry Facility, Harvard Medical School). This analysis suggested that one phosphorylated residue was present among the three amino acids closely situated at positions 11–14 of the N-terminus of L13. Among these, T12 is absolutely conserved in all available bacterial L13 protein sequences, whereas T11 and S14 are exclusively conserved across the *Mycobacterium* genus (Fig 5A, marked with asterisks). We first created a triple mutant of *Mtb* L13, termed L13(3A), in which all three residues T11, T12 and S14 were mutated to alanine. *In vitro* phosphorylation assays showed that this mutant protein was no longer phosphorylated by PknG (Fig 5B, lane 5), confirming that the phosphorylated amino acid is among these three residues. Next, mutant L13 proteins with single mutations were made and the purified proteins were individually re-tested in *in vitro* phosphorylation assays. Whereas L13(T12A) and L13(S14A) mutants were readily phosphorylated (Fig 5B, lanes 7 and 8), L13(T11A) completely failed to be phosphorylated by PknG (Fig 5B, lane 6), similar to the triple mutant L13(3A) (Fig 5B, lane 5). These results indicate that the mycobacterial conserved T11 of L13 is uniquely phosphorylated by PknG.

To assess if the phosphorylation status of L13 at T11 plays a role in the biofilm growth modulated by PknG, mutant alleles *rplM* ^*T11A*^ and *rplM* ^*T11E*^ were used to replace wild type *rplM* in *M*. *smegmatis* genomes. Whereas L13(T11A) is not activated by PknG, L13(T11E) mimics the conformation of phosphorylated L13. In *M*. *smegmatis*, replacement was successful with either *rplM* ^*T11A*^ or *rplM* ^*T11E*^ allele (Fig 5C). In *Mtb*, however, we were only able to create a *Mtb*.*L13(T11A)* replacement mutant (*rplM* ^*T11A*^) but failed to obtain *Mtb*.*L13(T11E)* (*rplM* ^*T11E*^). These results may suggest the essentiality of the non-phosphorylated form of L13 in *M*. *tuberculosis*, as indicated by previous work [19].

The *Ms*.*L13(T11A)* mutant displayed biofilm growth defects, replicating the phenotypes observed previously with *Ms*Δ*pknG* and *Ms*Δ*renU* (Fig 5D–5E), whereas the *Ms*.*L13(T11E)* mutant just showed a minor reduction compared to wild type (Fig 5D–5E, far right panel). *In trans* expression of the *L13(T11E)* allele from an integrative vector restored biofilm growth to the *Ms*.*L13(T11A)* mutant. Similar to *M*. *smegmatis*, the *Mtb*.*L13(T11A)* exhibited biofilm growth defects that could be rescued by *in trans* expression of an *L13(T11E)* allele (Fig 5F–5G). Unlike wild type *Mtb* or the *Mtb*Δ*pknG*/*pknG* strains (Fig 1F), the PknG inhibitor AX20017 failed to block biofilm growth of the *Mtb*.*L13(T11A)*/*(T11E)* strain (Fig 5F–5G). Together, these observations suggest that (i) phosphorylation of L13 by PknG is required for mycobacterial biofilm growth and that (ii) PknG, L13, and RenU form a functional cascade that modulates this static growth type in mycobacteria.

### The PknG-L13-RenU Axis Senses and Regulates Cellular NADH Level

**Fig 6:**
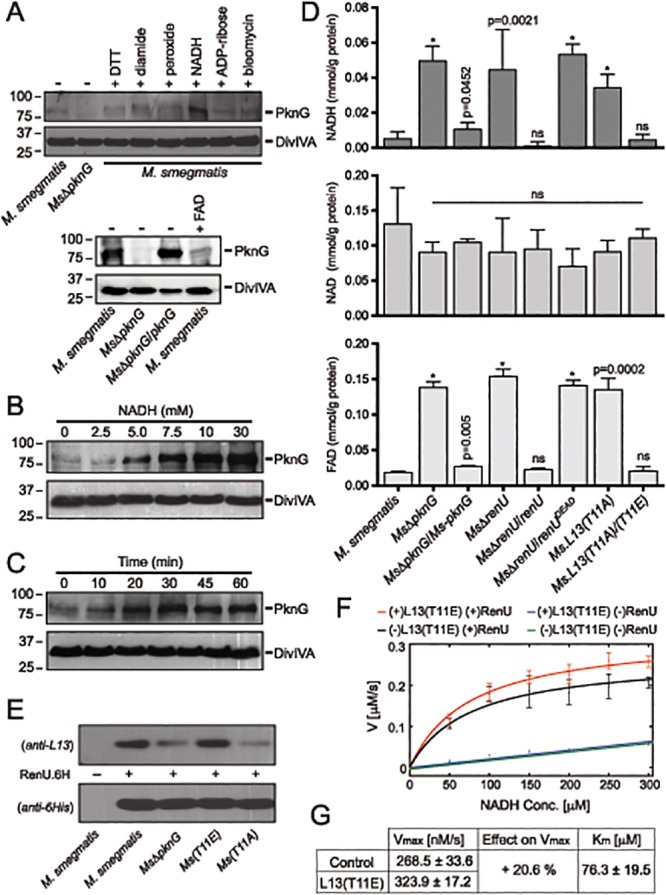
Correlation of NADH and RHOCS, and role of L13 phosphorylation by PknG. (**A**) Induction of PknG expression in *M*. *smegmatis*. Western analysis was used to detect PknG expression following the exposure of wild type *M*. *smegmatis* cultures (OD_600_ of 2) to various oxidative stimuli including NADH (upper) and FAD (lower) for 30 minutes. All chemicals were used at 10 mM except for bleomycin, which was used at 10 μg/ml. Samples were separated on SDS-PAGE, followed by immunodetection using an anti-PknG antibody or an anti-DivIVA antibody, as a control. Non-induced lysates from wild type *M*. *smegmatis* and *Ms*Δ*pknG* were used as controls. NADH uniquely induced expression of PknG. (**B**) Titration of the induced PknG expression by increasing NADH concentrations (0–30 mM) for 30 minutes, followed by Western analysis using anti-PknG antibody. (**C**) Time course of PknG expression (0–60 minutes) following cell exposure to 10 mM NADH. Detection of PknG was similar to (A) and (B). (**D**) Quantitation of cellular NADH (top), NAD^+^ (middle), and FAD (bottom) levels following oxidative stress induced by H_2_O_2_. *M*. *smegmatis* cells were exposed to 1 mM H_2_O_2_ for 1 hour. Bars show means with standard deviations from 3–6 biological repeats. *, p < 0.0001; ns, not significant relative to wild type *M*. *smegmatis*). (**E**) Effect of PknG-catalyzed phosphorylation of L13 on its association with RenU in the cytoplasm. Expression of PknG in *M*. *smegmatis* strains was induced by NADH. Cells were disintegrated by French Press, followed by ultracentrifugation to remove ribosomes. RenU.6H was added to the non-ribosomal fraction, followed by pull-down using Cobalt-agarose beads. The presence of L13 in the pulled down materials was detected by Western analysis using anti-L13 antibody. (**F**) Effect of L13(T11E), a phosphorylation-mimic form of L13, on *in vitro* NADH hydrolytic activity of RenU. Initial rates from a continuous fluorescence excitation assay were fit by nonlinear least squares to the Michaelis-Menten equation to determine K_m_ and V_max_ values for RenU. Reaction was performed at 37°C. Error bars represent standard deviations of triplicates. The extent of the uncatalyzed reaction was ~10% of the RenU catalyzed reaction. (**G**) Effect of L13(T11E) on the catalytic activity of RenU. In the presence of L13(T11E), a 20.6% increase in V_max_ was observed (p < 0.05x10^-3^), whereas K_m_, reflecting the binding affinity of RenU to NADH, was not affected by L13(T11E).

A recent study showed that expression of PknG is tightly regulated by unknown mechanisms related to the pathogenicity of *Mtb* [20]. Whereas PknG is highly expressed in slow growing mycobacteria such as *Mtb* and *M*. *bovis* BCG, the expression in *M*. *smegmatis* is extremely low [4,20]. To investigate the conditions that trigger PknG expression in *M*. *smegmatis*, the bacterium was treated with various redox stimuli, followed by analysis of PknG levels by Western analysis using a specific polyclonal antibody [3,4,20]. Interestingly, we found that PknG expression is uniquely induced when *M*. *smegmatis* cells are exposed to high levels of NADH (Fig 6A). None of the other tested chemicals, including FAD (lower panel), induced PknG expression. The induction of PknG expression by NADH, in both concentration- (Fig 6B) and time-dependent (Fig 6C) manners, may suggest a specific regulatory mechanism, similar to the Rex system originally described in *Streptomyces* [21–24], or an indirect effect due to changes in cellular metabolism or physiology caused by NADH exposure.

The facts that (i) PknG expression is induced by NADH (Fig 6A–6C), (ii) RenU preferentially degrades this redox cofactor *in vitro* (Fig 3A–3C), and (iii) absence of PknG or RenU leads to failed oxidative stress responses (Fig 2B–2C), indicate that the RHOCS pathway involving PknG, L13, and RenU regulates cellular redox homeostasis through an NAD(H)-related mechanism. To elucidate if interruption of RHOCS activities affects cellular NADH levels, *M*. *smegmatis* mutants of the PknG-L13-RenU axis and the parental strain mc^2^155 were challenged with H_2_O_2_, followed by extraction and analysis of NADH, NAD^+^, and FAD concentrations. Whereas interruption of RHOCS did not affect NAD^+^ level, it resulted in dramatic accumulations of NADH and FAD by H_2_O_2_ (Fig 6D). These observations reveal a novel mechanism of redox homeostasis that senses and regulates the cellular levels of NADH, the possibly other nucleoside diphosphate derivatives including FAD.

### Phosphorylation by PknG Promotes the Cytoplasmic Association of L13 with RenU and Its NADH Hydrolytic Activity

To better understand the role of the phosphorylation of L13 by PknG, we first analyzed if phosphorylation affects the formation of the L13-RenU complex in the mycobacterial cytoplasm. *M*. *smegmatis* strains representing different states of L13 phosphorylation were first exposed to NADH to induce PknG expression. Cell lysates were prepared and ribosomes removed by ultracentrifugation. RenU.6H was then added to the non-ribosomal fractions. After incubation, RenU.6H was purified using Cobalt agarose beads and the co-purification of L13 analyzed by Western analysis using a polyclonal anti-L13 antibody. Whereas RenU.6H was equally detected, L13 association with RenU.6H in the cytoplasm was dependent on its phosphorylation by PknG (Fig 6E). This experiment suggests that phosphorylation of L13 at T11 by PknG promotes its association with RenU in the mycobacterial cytoplasm.

Because the function of RHOCS is involved with regulation of cellular NADH levels (Fig 6A–6D), we examined if phosphorylated L13 affects the RenU-catalyzed NADH hydrolysis (Fig 3C). To study this question, we first established a fluorescence-based assay that allowed continuous monitoring of NADH hydrolysis by RenU. The assay was based on the different spectral characteristics of folded and unfolded conformations of NADH in aqueous solutions as previously reported [25]. NADH absorbs light at a wavelength of 260 nm through its adenine moiety and emits light at a wavelength of 460 nm through its nicotinamide moiety. The efficiency of the energy transfer responsible for this excitation/emission characteristic is decreased in the unfolded conformation or, in the case of this assay, upon the hydrolysis of NADH.

The hydrolysis of NADH by RenU in the presence or absence of L13(T11E), a mimic form of phosphorylated L13, was monitored. The initial rates were fit to the Michaelis-Menten equation (Fig 6F) to determine K_m_ and V_max_ values (Fig 6G). Analysis of K_m_ confirmed that there was no effect of L13(T11E) on RenU’s NADH-binding affinity (one-way ANOVA, effect of L13(T11E), F(3,8) = 2.06, p>0.18) (Fig 6G). In addition, L13(T11E) did not have observable effects on NADH hydrolysis in the absence of RenU (Fig 6F, blue vs green); and wild type L13 did not have observable effects on the NADH hydrolysis catalyzed by RenU (S7 Fig, panel A). Importantly, analysis of V_max_ of the reactions performed at 37°C displayed a 20.6% increase in the rate of NADH hydrolysis in the presence of L13(T11E) (one-way ANOVA, effect of L13(T11E), F(3,8) = 35.80, p<0.05×10^-3^) (Fig 6F, red vs black, and 6G). An increase in V_max_ was also observed with temperature increments up to 42°C (S7 Fig, panel B). Together, these data suggest that the phosphorylation of L13 by PknG directly impacts not only the association of L13 with RenU in the mycobacterial cytoplasm (Fig 6E), but also the NADH hydrolytic activity catalyzed by RenU (Fig 6F–6G).

### RHOCS Is Required for Survival of *M*. *tuberculosis* in Host Macrophages

**Fig 7:**
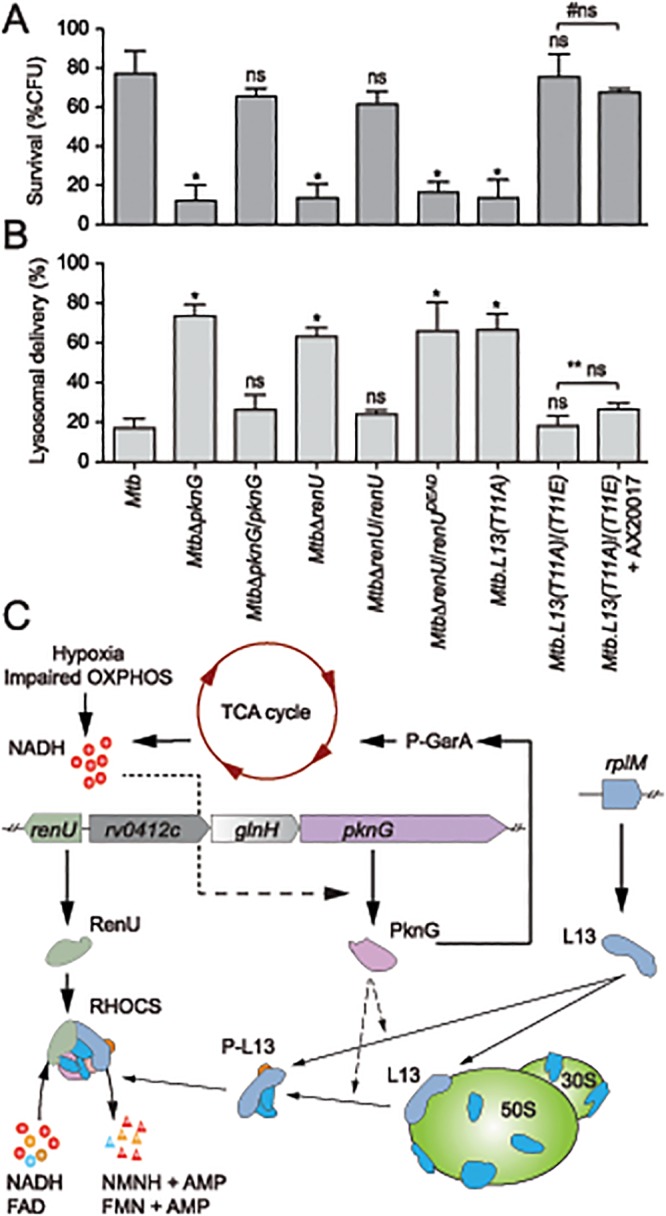
Intracellular trafficking and survival of *M*. *tuberculosis* RHOCS mutants. (**A**) Intracellular survival of *Mtb* strains. Macrophages were infected with *Mtb* strains for 3 hours, followed by 0 or 72-hour chase. CFUs were counted after 4–5 weeks of growth at 37°C. Bars represent percentages of CFUs remaining at 72-hour compared to 0-hour time point. Error bars represent standard deviations from 3–6 repeats. *, p < 0.001; ns, not significant relative to *Mtb* H37Rv; #ns, not significant between the two indicated groups. Order of strains is as in 7B. (**B**) Quantitative analysis of lysosomal delivery following phagocytosis of *Mtb* strains by macrophages. Macrophages were infected with FLUOS-stained *Mtb* strains for 1 hour, followed by 16-hour chase. Infected macrophages (see C below) were used for quantitation. Biological triplicates of 50 events were counted for each *Mtb* strain. Error bars represent standard deviations. Error bars represent standard deviations from 3–6 repeats. *, p < 0.0001; ns, not significant relative to *Mtb* H37Rv; ** ns, not significant between the two indicated groups. (**C**) A model depicting activity and function of RHOCS in mycobacteria. PknG was previously shown to de-repress the TCA cycle through its phosphorylation of GarA, an inhibitor of α-ketoglutarate decarboxylase and glutamate dehydrogenase. Increased TCA cycle activities, hypoxia, or impaired oxidative phosphorylation (OXPHOS), lead to elevated NADH levels. To protect mycobacterial cells against the change in redox status, PknG expression is up-regulated, leading to the signaling cascade including L13 and RenU, which degrades NADH and FAD and restores their optimal level. AMP, adenosine monophosphate, FAD, flavin adenine dinucleotide; FMN, flavin mononucleotide; NMNH, nicotinamide mononucleotide.

To investigate if RHOCS is related to the role of PknG in the survival and lysosomal delivery of pathogenic mycobacteria in infected macrophages [3], *Mtb* RHOCS mutants and the parental strain H37Rv were used to infect murine bone-marrow derived macrophages. Survival was determined by measuring colony forming units (CFUs) of internalized *Mtb* cells at day 0 and day 4 following the infection. As shown in Fig 7A, deletion of *pknG* or *renU*, or the single mutation *L13(T11A)*, significantly reduced the percentages of *Mtb* survival at day 4 relative to day 0. By contrast, the complemented strains, namely *Mtb*Δ*pknG*/*pknG*, *Mtb*Δ*renU*/*renU*, and *L13(T11A)*/*(T11E)*, showed survival similar to wild type *Mtb* (Fig 7A). As shown with biofilm growth, the mutant allele r*enU* ^*DEAD*^ failed to restore the survival of the *Mtb*Δ*renU* mutant (Fig 7A). In addition, *in trans* expression of the phosphorylation-mimic form of L13, L13(T11E), also made the *L13(T11A)*/*(T11E)* strain resistant to AX20017, the PknG specific inhibitor. These results, all together, indicated that the kinase activity of PknG, the Nudix hydrolase activity of RenU, as well as the phosphorylation of L13, each is required for the survival of *Mtb* in host macrophages.

Next, to analyze if the intracellular survival of *Mtb* strains correlated with their lysosomal delivery levels, trafficking of internalized *Mtb* strains was analyzed by microscopy. As shown previously [3], absence of *pknG* resulted in increased lysosomal delivery (Fig 7B). The *Mtb*Δ*pknG* mutant was largely localized within acidic milieus, whereas wild type *Mtb* displayed a low level of lysosomal localization (Fig 7B). *In trans* expression of *pknG* restored wild type level of lysosomal delivery to *Mtb*Δ*pknG* (Fig 7B). In addition, *Mtb*Δ*renU* and *Mtb*.*L13(T11A)* mutants exhibited lysosomal delivery levels comparable to that of *Mtb*Δ*pknG* (Fig 7B) while the corresponding complemented strains, *Mtb*Δ*renU*/*renU* and *L13(T11A)*/*(T11E)*, behaved like wild type in lysosomal delivery. In an agreement with the survival (Fig 7A), RenU^DEAD^ failed to rescue *Mtb*Δ*renU* and the *L13(T11A)*/*(T11E)* strain showed resistance to AX20017 (Fig 7A). These results suggest that the function of RHOCS in *Mtb* lysosomal delivery is correlated, either as a cause or a consequence, to the survival of the bacillus in the macrophage.

## Discussion

This work has revealed a novel signaling mechanism that is used by mycobacteria to regulate cellular redox homeostasis (Fig 7C). We propose that this system, RHOCS, is capable of sensing the key redox regulator NADH, and regulating its cellular level through direct degradation. RHOCS is composed of at least three components: a eukaryotic-type protein kinase, PknG, a ribosomal protein, L13, and a Nudix hydrolase, RenU. RHOCS is responsive to cellular NADH levels through up-regulated expression of PknG, which phosphorylates L13 at a unique site, T11, and promotes its cytoplasmic association with RenU. At least two possible mechanisms may lead to this increased L13-RenU association: (i) phosphorylation of L13 by PknG prevents the association of L13 with the ribosome, or (ii) the phosphorylation causes releases of L13 from the ribosome, similar to the effect of L13a phosphorylation by ZIPK observed in human macrophages [26]. Once associated with RenU in the cytoplasm, phosphorylated L13 accelerates the Nudix hydrolase activity of RenU that directly degrades NADH, thus lowering its cellular level. This paradigm of redox regulation is novel and has not been observed before in bacteria.

Despite its localization on the large ribosomal subunit, the canonical ribosomal function of L13 remains enigmatic. *E*. *coli* L13 was suggested to contribute to the first step of 50S subunit assembly [27], whereas the human analog L13a might play a role in ribosomal RNA methylation [28]. By contrast, accumulating evidence suggests that L13 plays several functions outside the ribosome. In *E*. *coli*, L13 forms a transcription anti-termination complex with other ribosomal proteins (RpL3, RpL4 and RpS4), which binds RNA polymerase and acts on Rho-dependent anti-terminators of ribosomal RNA [29]. L13 was also shown to interact with Obg, an essential GTP binding protein involved in growth promotion and stress response in *B*. *subtilis* [30]. In human macrophages, treatment with interferon-γ (IFN-γ) induces phosphorylation of L13a by ZIPK [26]. Phosphorylated L13a is then released from the ribosome to form, with four other proteins, the IFN-*G*amma-*A*ctivated *I*nhibitor of *T*ranslation (GAIT) complex that binds messenger RNAs and inhibits translation of proteins involved in IFN-γ responses [26,31]. Our data reveal a novel extra-ribosomal function of L13, triggered through its phosphorylation by PknG, in redox homeostatic regulation in mycobacteria. The fact that both *Mtb* and its host macrophage use L13 phosphorylation as a common method to convey cellular stress responses is fascinating and warrants further investigation. Our work also supports the recent “depot hypothesis”, which proposes that macromolecular complexes such as the ribosome function as “reservoirs” for regulatory proteins that perform non-canonical functions [32,33].

The mechanism of action proposed for RHOCS (Fig 7C) fits nicely with some of the previous observations. Work by other groups suggests that PknG derepresses the TCA cycle through its phosphorylation of the cycle inhibitors GarA or OdhI [34,35]. Thus, activity of PknG in the TCA cycle is expected to increase production of NADH, which is then fed into the oxidative phosphorylation pathway that produces reactive oxygen species and free radicals. In addition, NADH is an effective inhibitor of α-ketoglutarate dehydrogenase, the key generator of NADH and oxidative stress [36], and a target of GarA [34,35]. Therefore, the role of PknG in the RHOCS pathway may provide mycobacteria with a supportive mechanism that prevents cell death from redox disturbance caused by increased TCA activity. Interestingly, free radicals produced from NADH by the TCA cycle were recently suggested to mediate bacterial cell death triggered by bactericidal antibiotics [37]. Accordingly, the function of RHOCS in cellular NADH regulation may also help to explain the recent observation that absence of PknG leads to enhanced antibiotic susceptibility in mycobacteria [4,38].

Bacterial responses to oxidative stress have emerged as an integral part of the developmental program required for biofilm growth [39–41]. For example, oxidative stress was shown to evoke metabolic adaptation that reduce NADH production [42] and induces biofilm formation [43]. Similarly, the transcription regulator SoxR, which helps bacteria to defend oxidative stress, was shown to coordinate biofilm growth in *Pseudomonas sp*., *E*. *coli*, and *S*. *coelicolor* [40,44]. We propose that RHOCS, through its role in redox stress regulation, is required for growth of mycobacteria in biofilms and the phagosomal milieu of host macrophages. The ability to sense and regulate cellular levels of NADH allows RHOCS to switch cells between different states of metabolism and physiology. Therefore, RHOCS may function as a key regulator that links redox homeostasis with the pathogenicity of *M*. *tuberculosis*.

## Materials and Methods

### Bacterial Strains and Growth Media

*M*. *tuberculosis* H37Rv, *M*. *bovis* BCG Pasteur, and *M*. *smegmatis* mc^2^155 (American Type Culture Collection) were used as parental strains. Mycobacterial strains were grown at 37°C in 7H10 or 7H9 with appropriate supplements and antibiotics. Kanamycin and hygromycin were used at 50 and 75 μg/ml, respectively. Biofilm growth was done as previously described [10] using Sauton’s medium. For quantitation, a syringe connected to a sterile needle was used to remove the liquid medium and planktonic cells beneath the films. The biomass was harvested and growth estimated through determination of total protein by Bradford method.

### Strain and Plasmid Construction

Targeted gene deletion or replacement was done by homologous recombination methods as previously reported [3,4]. Details can be found in the S1 Text. Plasmids and oligonucleotides used in this study can be found in S4 and S5 Tables, respectively.

### Intracellular Survival of *M*. *tuberculosis* Strains

Macrophages, generated as described in Extended Experimental Procedures, were seeded in 12-well tissue culture plates (BD Biosciences, San Jose, CA) and let adhere overnight (37°C, 10% CO_2_) prior to infection. *Mtb* strains were grown to saturation and infections were performed at MOI 50:1 for 3 hours. Infected macrophages were washed with warm PBS and incubated for 45 minutes with 200 μg/ml amikacin to kill extracellular bacteria, and exchanged into fresh DMEM. At 0 or 72 hours of incubation at 37°C and 10% CO_2_, infected macrophages were processed for CFU assays by washing 3 times with PBS, followed by lysis of the macrophages by 0.05% SDS for 5 minutes. Supernatants were harvested, vortexed thoroughly, and plated in triplicate in ten-fold dilutions onto 7H10-OADC agar. Plates were incubated at 37°C for 4 to 5 weeks before CFUs were counted.

### Statistical Analysis

Statistical analyses were conducted using GraphPad Prism 5.0f software (La Jolla, CA). Students two-tailed *t*-test was used to analyze the statistical significance of differences between groups. For enzyme kinetics experiments, one-way analysis of variance was performed using Excel, and non-linear least squares fits were conducted in Matlab by Mathworks (Natick, MA).

## Supporting Information

S1 Fig PknG is required for biofilm growth in *M*. *bovis* BCG. Biofilm growth of wild type *M*. *bovis* BCG Pasteur and its derived BCGΔ*pknG* mutant. Pictures were taken after 5 weeks of growth at static humidified condition of 37°C and 5% CO_2_. Shown images are representatives of biological triplicates.
(PDF)

S2 Fig Surface attachment of *M*. *smegmatis* strains. (**A**) Attachment of wild type *M*. *smegmatis* mc^2^155 and its derived *Ms*Δ*pknG* mutant, both of which express a green fluorescent protein, to a PVC surface. Attachment was recorded at day 3, 5, and 7 after inoculation. (**B**) Uneven attachment of *Ms*Δ*pknG* cells to the PVC surface at day 7, illustrated in the Z sections derived from confocal microscopy. Horizontal axis indicates the approximate position of the surface while vertical axis shows the direction of biofilm growth away from the surface.
(PDF)

S3 Fig Effect of redox environments on PknG kinase activities. PknG was incubated for 30 min at 37°C in kinase reaction buffer containing 10 μCi of [γ-^32^P]-ATP and varied concentrations of DTT. Reactions were performed in the absence (**A**) or presence (**B**) of L13 as substrate. Samples were electrophoresed on a 15% SDS-PAGE gels, transferred onto PVDF membranes, and followed by autoradiography (upper panels) or Coomassie Blue staining (lower panels).
(PDF)

S4 Fig Oligomerization state of RenU. Size exclusion chromatography on a Sephacryl 16/60 S-200 column was conducted to determine the oligomerization state of RenU.6H. Shown are elution profiles of RenU.6H (solid red line) and proteins of known oligomerization and molecular weights as standards. RenU.6H (~17 kDa) was separated between cytochrome C (12.4 kDa) and carbonic anhydrase (29 kDa), indicating a monomeric state in solution.
(PDF)

S5 Fig Substrate specificity of RenU. (**A**) The relative activity was calculated from the measurements of phosphomolybdate absorbance at 820 nm. The data were normalized to the highest measurement. RenU was most active against ADP-Ribose, the only NDPX assayed, compared to the panel of NTPs tested. (**B**) Additional RenU substrate specificity. The relative activity was calculated from measurements of phosphomolybdate absorbance at 820 nm. The data were normalized to the highest measurement. FAD, ADP-Ribose, and NADH exhibit the most hydrolysis by RenU compared to the other NDPXs tested.
(PDF)

S6 Fig Kinetics of RenU with its three preferred substrates. Kinetic characterization of the top three substrates of RenU. Initial rates from a discontinuous colorimetric assay for NADH (left), FAD (center), and ADPR (right) were fit by nonlinear least squares to the Michaelis-Menten equation.
(PDF)

S7 Fig Effect of L13 phosphorylation on *in vitro* NADH hydrolysis by RenU. (**A**) Effect of wild type L13 on NADH hydrolysis by RenU. Initial rates from a continuous fluorescence excitation assay were fit by nonlinear least squares to the Michaelis-Menten equation. Error bars represent standard deviations of triplicates. (**B**) Effect of L13(T11E) on the RenU-catalyzed NADH hydrolysis at different temperatures. Initial rates from a continuous fluorescence excitation assay were fit by nonlinear least squares to the Michaelis-Menten equation. Error bars represent standard deviations of triplicates.
(PDF)

S1 Table Surface properties of *M*. *smegmatis* strains. (DOCX)

S2 Table Chemically synthesized DNA sequence for expression of RenU^DEAD^. (DOCX)

S3 Table RenU co-purified proteins identified by LC/MS/MS. (DOCX)

S4 Table Plasmids and phasmids used in this study. (DOCX)

S5 Table Primers used in this study. (DOCX)

S1 Text Additional methods used in this study. (DOCX)
