# Supplementary figures and images for: A Redox Regulatory System Critical for Mycobacterial Survival in Macrophages and Biofilm Development
Source: PLoS Pathog. 2015 Apr 17;11(4):e1004839. doi: 10.1371/journal.ppat.1004839 (PMC4401782; doi:10.1371/journal.ppat.1004839)

*M. bovis* BCG

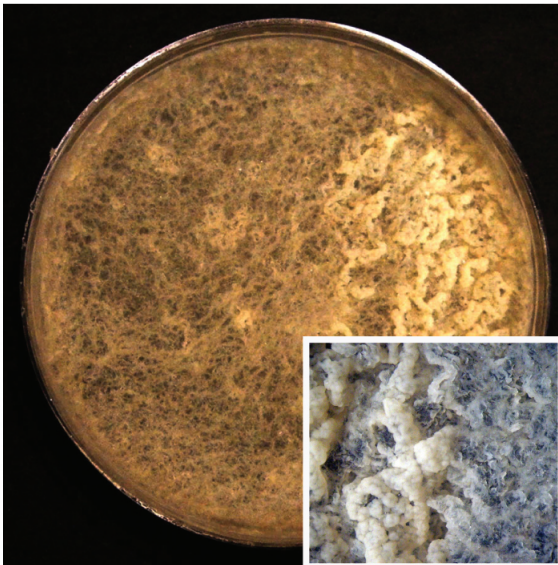

BCG $\Delta$ *pknG*

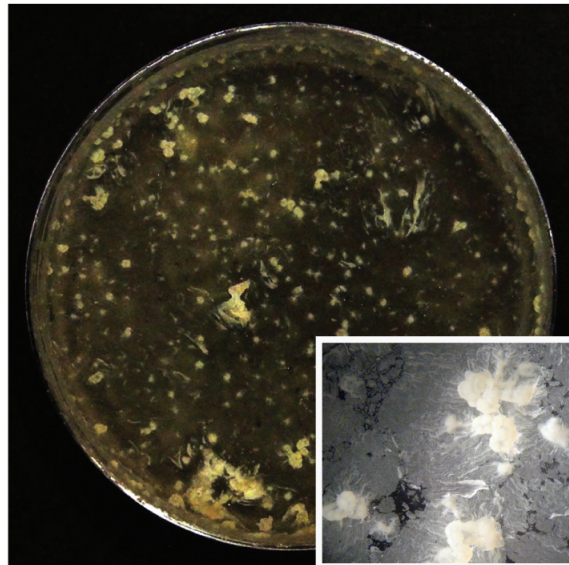

Supplement: S1 Fig — Biofilm growth of wild type M. bovis BCG Pasteur and its derived BCGΔpknG mutant. Pictures were taken after 5 weeks of growth at static humidified condition of 37°C and 5% CO2. Shown images are representatives of biological triplicates. (PDF) [file ppat.1004839.s001.pdf]

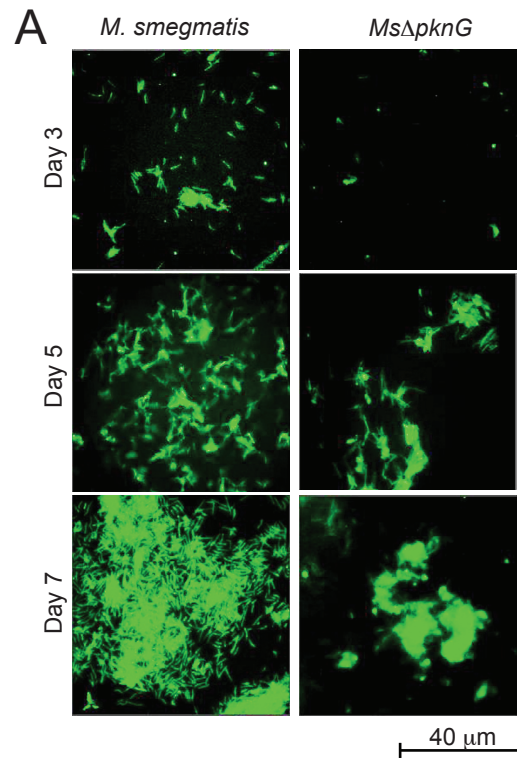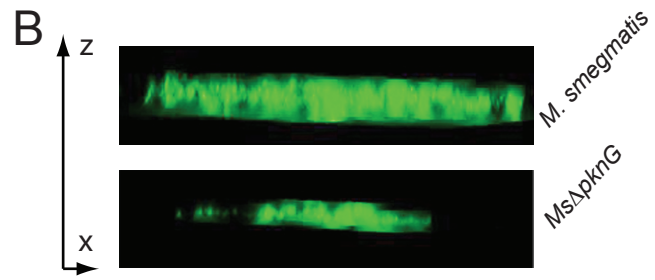

Supplement: S2 Fig — (A) Attachment of wild type M. smegmatis mc2155 and its derived MsΔpknG mutant, both of which express a green fluorescent protein, to a PVC surface. Attachment was recorded at day 3, 5, and 7 after inoculation. (B) Uneven attachment of MsΔpknG cells to the PVC surface at day 7, illustrated in the Z sections derived from confocal microscopy. Horizontal axis indicates the approximate position of the surface while vertical axis shows the direction of biofilm growth away from the surface. (PDF) [file ppat.1004839.s002.pdf]

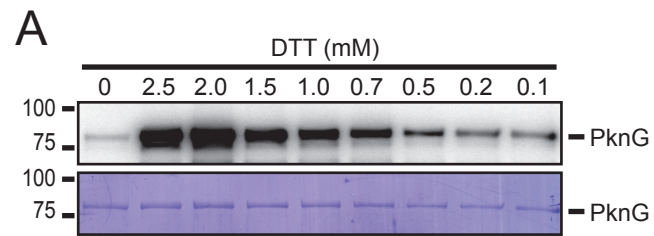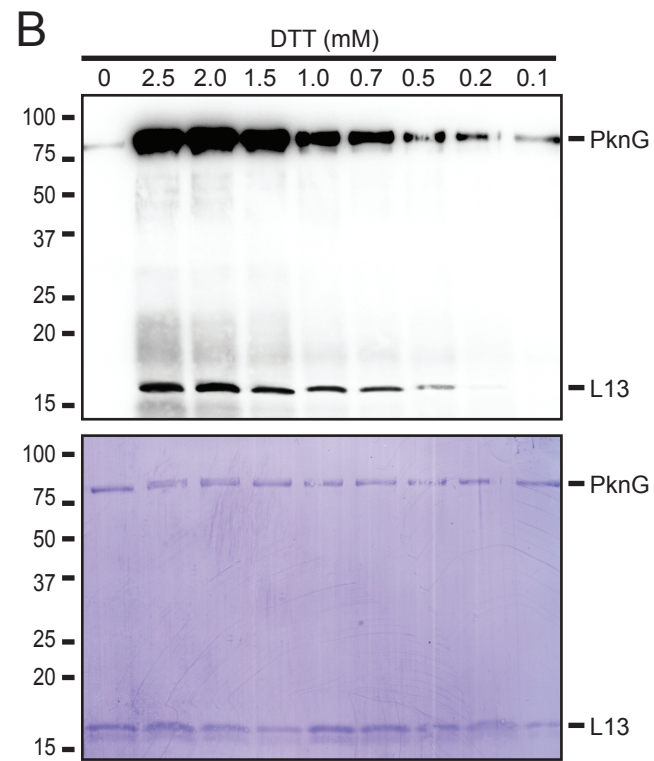

Supplement: S3 Fig — PknG was incubated for 30 min at 37°C in kinase reaction buffer containing 10 μCi of [γ-32P]-ATP and varied concentrations of DTT. Reactions were performed in the absence (A) or presence (B) of L13 as substrate. Samples were electrophoresed on a 15% SDS-PAGE gels, transferred onto PVDF membranes, and followed by autoradiography (upper panels) or Coomassie Blue staining (lower panels). (PDF) [file ppat.1004839.s003.pdf]

### HiPrep Sephacryl S-200 Gel Filtration Chromatography

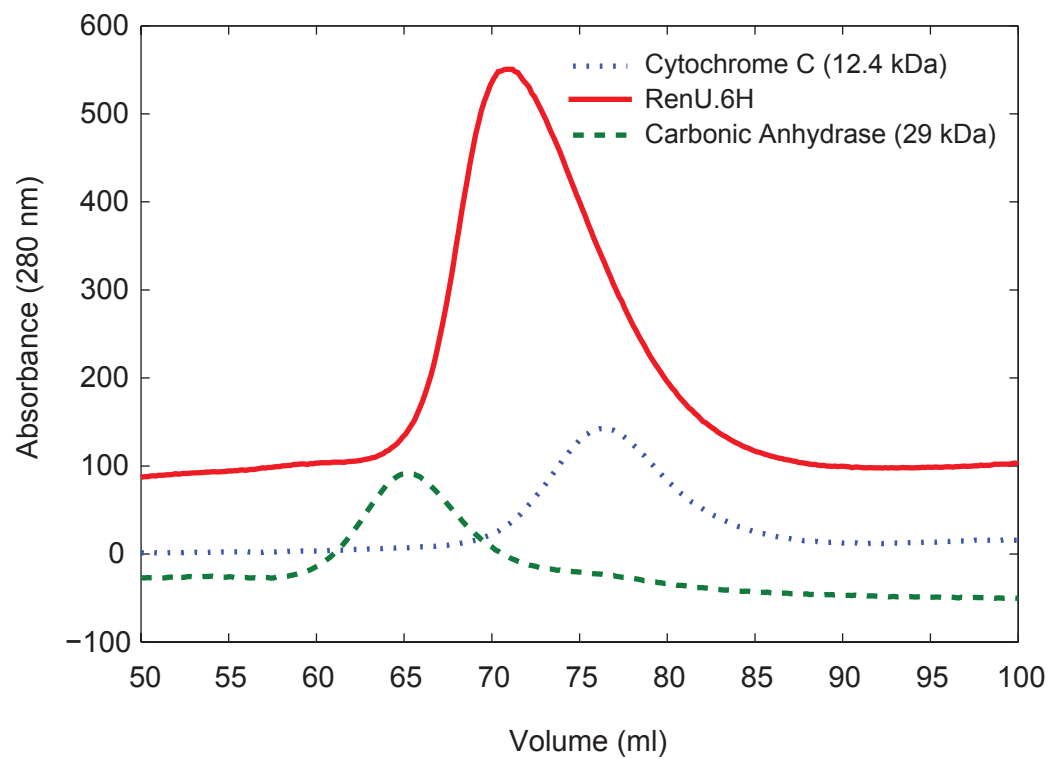

Supplement: S4 Fig — Size exclusion chromatography on a Sephacryl 16/60 S-200 column was conducted to determine the oligomerization state of RenU.6H. Shown are elution profiles of RenU.6H (solid red line) and proteins of known oligomerization and molecular weights as standards. RenU.6H (~17 kDa) was separated between cytochrome C (12.4 kDa) and carbonic anhydrase (29 kDa), indicating a monomeric state in solution. (PDF) [file ppat.1004839.s004.pdf]

**A**

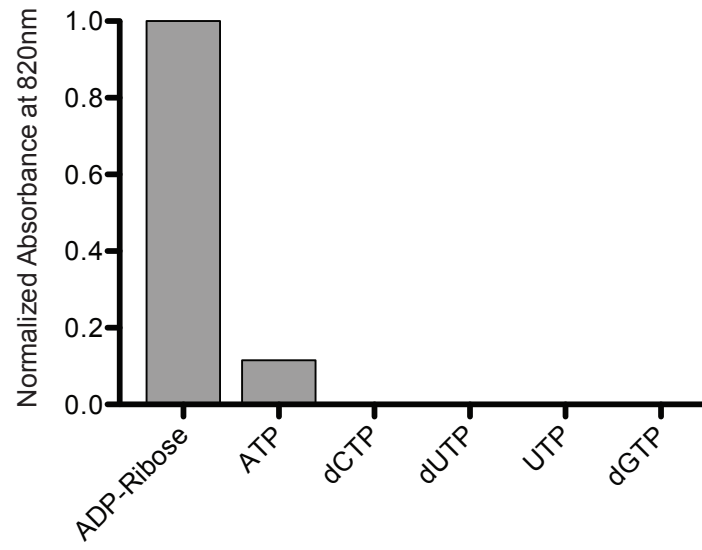

**B**

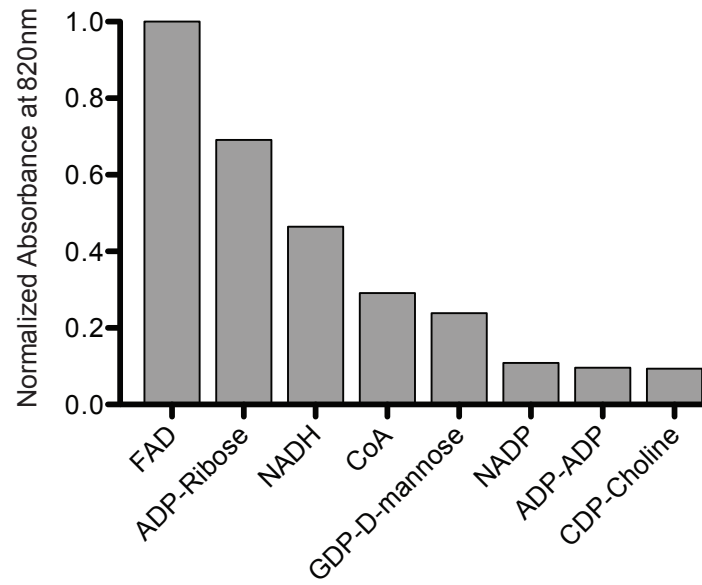

Supplement: S5 Fig — (A) The relative activity was calculated from the measurements of phosphomolybdate absorbance at 820 nm. The data were normalized to the highest measurement. RenU was most active against ADP-Ribose, the only NDPX assayed, compared to the panel of NTPs tested. (B) Additional RenU substrate specificity. The relative activity was calculated from measurements of phosphomolybdate absorbance at 820 nm. The data were normalized to the highest measurement. FAD, ADP-Ribose, and NADH exhibit the most hydrolysis by RenU compared to the other NDPXs tested. (PDF) [file ppat.1004839.s005.pdf]

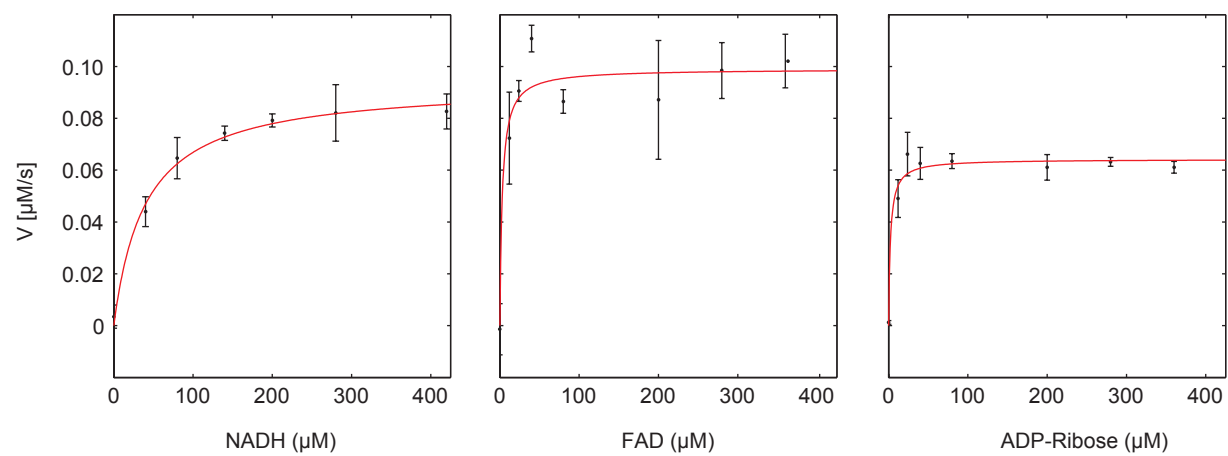

Supplement: S6 Fig — Kinetic characterization of the top three substrates of RenU. Initial rates from a discontinuous colorimetric assay for NADH (left), FAD (center), and ADPR (right) were fit by nonlinear least squares to the Michaelis-Menten equation. (PDF) [file ppat.1004839.s006.pdf]

**A**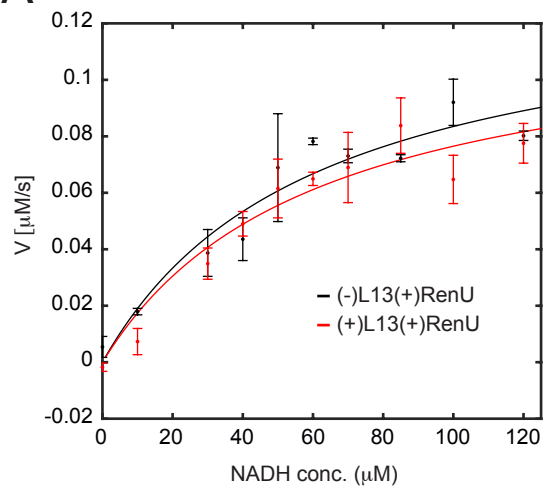**B**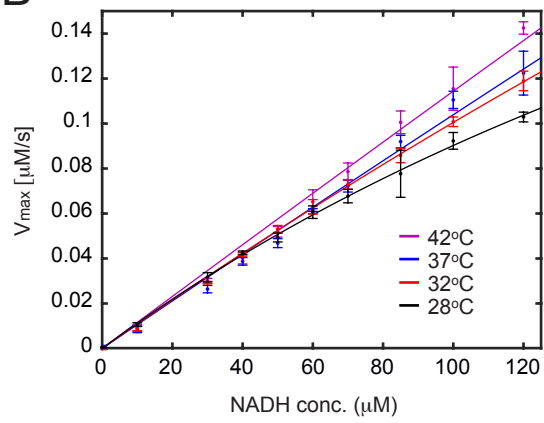

Supplement: S7 Fig — (A) Effect of wild type L13 on NADH hydrolysis by RenU. Initial rates from a continuous fluorescence excitation assay were fit by nonlinear least squares to the Michaelis-Menten equation. Error bars represent standard deviations of triplicates. (B) Effect of L13(T11E) on the RenU-catalyzed NADH hydrolysis at different temperatures. Initial rates from a continuous fluorescence excitation assay were fit by nonlinear least squares to the Michaelis-Menten equation. Error bars represent standard deviations of triplicates. (PDF) [file ppat.1004839.s007.pdf]
