# Supplementary material for: A Redox Regulatory System Critical for Mycobacterial Survival in Macrophages and Biofilm Development
Source: PLoS Pathog. 2015 Apr 17;11(4):e1004839. doi: 10.1371/journal.ppat.1004839 (PMC4401782; doi:10.1371/journal.ppat.1004839)
Supplement: S1 Table — (DOCX) [file ppat.1004839.s008.docx]

| **Surface property**  **(mean ± SD)** | **Strain** | | |
| --- | --- | --- | --- |
|  | ***M. smegmatis*** | ***Ms*∆*pknG*** | ***Ms*∆*pknG*/*Ms pknG*** |
| Cell-cell aggregation^1^ | 18.8 ± 3.6 | 72.0 ± 2.5 | 24.7 ± 1.3 |
| Hydrophobicity index^2^ | 27.3 ± 2.41 | 13.6 ± 5.34 | 29.3 ± 2.96 |
| Congo red binding^3^ | 22.4 ± 4.5 | 11.9 ± 1.6 | 25.7 ± 4.8 |
| Zeta potential^4^ | -20.5 ± 1.26 | -27.1 ± 1.57 | -23.7 ± 0.71 |

^1^Percentage of aggregated cells. ^2^Arbitrary units, percentage of cells recovered in

organic phase. ^3^Cell wall-bound Congo red, OD_488_/g dry cell mass. ^4^Expressed in mV
