# Supplementary material for: A Redox Regulatory System Critical for Mycobacterial Survival in Macrophages and Biofilm Development
Source: PLoS Pathog. 2015 Apr 17;11(4):e1004839. doi: 10.1371/journal.ppat.1004839 (PMC4401782; doi:10.1371/journal.ppat.1004839)
Supplement: S2 Table — (DOCX) [file ppat.1004839.s009.docx]

| **> *renU*^DEAD^**  gaattccatatgcgtggcgacggagatggctgggtgatgtcggagaacggcgcccggttctggggtcgccacggcgcggccggtttgctgttgcgggccccgatgcccggcggcgcggcggcggtgctgttgcagcaccgcgcgccgtggagtcatcagggcggaacgtgggcgctgcccggcggtgcacgcgacagtcatgagacccctgagcaggccgcggtgcgcgcggcgcacgcagcggccggcctacccgccgagcaactgacggtgcgcacgacggtggtgaccgccgaggtcgcaggcatcggcggcacgcagtggacgtacacgacggtgatcgccgacgcggccgaacccctgcacaccgtgcccaaccgggagagcgccgagttgcgctgggtcctcgaggatcaggtcgcggacctgccgctgcatcccgggtttgccgcgagttggcagcgcctacgtgaggtgaccgcgacgatcccgttgctcaaccggcagcgctgatcggatccaagctt |
| --- |
| **> RenU^DEAD^**  MRGDGDGWVMSENGARFWGRHGAAGLLLRAPMPGGAAAVLLQHRAPWSHQGGTWALPGGARDSHETPEQAAVRAAHAAAGLPAEQLTVRTTVVTAEVAGIGGTQWTYTTVIADAAEPLHTVPNRESAELRWVLEDQVADLPLHPGFAASWQRLREVTATIPLLNRQR |
