# Supplementary material for: A Redox Regulatory System Critical for Mycobacterial Survival in Macrophages and Biofilm Development
Source: PLoS Pathog. 2015 Apr 17;11(4):e1004839. doi: 10.1371/journal.ppat.1004839 (PMC4401782; doi:10.1371/journal.ppat.1004839)
Supplement: S3 Table — (DOCX) [file ppat.1004839.s010.docx]

| **Protein** | **Gene** |
| --- | --- |
| Nudix Hydrolase protein, RenU | *msmeg_0790* |
| Cupin domain protein | *msmeg_5707* |
| 30S ribosomal protein S19, RpsS | *msmeg_1440* |
| Pseudouridine synthase, RluB | *msmeg_3740* |
| Urease accessory protein, UreE | *msmeg_1091* |
| Pantothenate kinase, CoaA | *msmeg_5252* |
| Transcriptional regulator | *msmeg_2153* |
| 50S ribosomal protein L27, RpmA | *msmeg_4624* |
| SsrA-binding protein, SmpB | *msmeg_2091* |
| Transposase | *msmeg_0073* |
| Transposase | *msmeg_0074* |
| Putative uncharacterized protein | *msmeg_4029* |
| 50S ribosomal protein L16, RplP | *msmeg_1443* |
| 50S ribosomal protein L13, RplM | *msmeg_1556* |
| 50S ribosomal protein L21, RplU | *msmeg_4625* |
