# Supplementary material for: A Redox Regulatory System Critical for Mycobacterial Survival in Macrophages and Biofilm Development
Source: PLoS Pathog. 2015 Apr 17;11(4):e1004839. doi: 10.1371/journal.ppat.1004839 (PMC4401782; doi:10.1371/journal.ppat.1004839)
Supplement: S4 Table — (DOCX) [file ppat.1004839.s011.docx]

| **Plasmid** | **Relevant Features** | **Ref** |
| --- | --- | --- |
| pCV125 | Kan^R^, mycobacterial integrative vector | [[1](#_ENREF_1)] |
| pMV361 | Kan^R^, mycobacterial integrative vector, P_hsp60_ promoter | [[2](#_ENREF_2)] |
| pVN747 | Hyg^R^, pMS2 derived replicative vector, P_SOD_ promoter | [[3](#_ENREF_3)] |
| pVN839 | Kan^R^, mycobacterial replicative vector, P_SOD_ | [9] |
| pPR27 | Gen^R^, mycobacterial replicative vector, temperature sensitive Ori, *sacB* | [[4](#_ENREF_4)] |
| pYUB854 | Hyg^R^, *E. coli* replicative vector, and lambda cos sites | [[5](#_ENREF_5)] |
| pPknG^K181M^ | Kan^R^, pSD5 derived, expressing *Mtb* PknG^K181M^ | [[6](#_ENREF_6)] |
| pVN578 | Kan^R^, pMV361 derived, expressing *Mtb* PknG from P_hsp60_ | [[7](#_ENREF_7)] |
| pVN579 | Kan^R^, pMV361-derived, expressing *M. smegmatis* PknG from P_hsp60_ | [[7](#_ENREF_7)] |
| pVN701B | Kan^R^ Gen^R^, pJV53 derived, ts-Ori, *sacB* , P_ace_-Che9c | [[7](#_ENREF_7)] |
| pVN740 | Hyg^R^, pYUB854 derived, AES for *pknG* deletion in *M. smegmatis* | [[7](#_ENREF_7)] |
| pVN753 | Kan^R^, pMV361 derived, expressing *M. smegmatis renU* from P_hsp60_ | [9] |
| pVN755 | Hyg^R^, pYUB854 derived, AES for deletion of *M. smegmatis renU* | [9] |
| pVN771 | Kan^R^, pMV361 derived, expressing *Mtb renU* from P_hsp60_ | [9] |
| pVN791 | Hyg^R^, pYUB854 derived, AES for deletion of *Mtb renU* | [9] |
| pVN792 | Amp^R^, pET15b derived, expressing *Mtb* 6H.PknG from P_T7_ | [9] |
| pVN823 | Hyg^R^, pVN747 derived, expressing *M. smegmatis* RenU.6H from P_SOD_ | [9] |
| pVN835 | Amp^R^, pET11c-derived, expressing *M. smegmatis* RenU.6H from P_T7_ | [9] |
| pVN840 | Kan^R^, pVN839 derived, expressing *Mtb* RenU from native promoter | [9] |
| pVN844 | Amp^R^, pET15b derived, expressing *M. smegmatis* 6H.L13 from P_T7_ | [9] |
| pVN866 | Kan^R^, pCV125 derived, expressing *M. smegmatis* RenU.6H from native promoter | [9] |
| pVN885 | Amp^R^, pET15b derived, expressing *Mtb* 6H.L13 from P_T7_ | [9] |
| pVN889 | Amp^R^, pET15b derived, expressing *Mtb* 6H.L13(3A) (L13^T11A,T12A,S14A^) from P_T7_ | [9] |
| pVN890 | Amp^R^, pET15b derived, expressing *Mtb* 6H.L13^T11A^ from P_T7_ | [9] |
| pVN891 | Amp^R^, pET15b derived, expressing *Mtb* 6H.L13^T12A^ from P_T7_ | [9] |
| pVN892 | Amp^R^, pET15b-derived, expressing *Mtb* 6H.L13^S14A^ from P_T7_ | [9] |
| pVN895 | Hyg^R^, pYUB854 derived, AES for replacement of *rplM* by *rplM^T11A^* in *M. smegmatis* | [9] |
| pVN896 | Hyg^R^, pYUB854 derived, AES for replacement of *rplM* by *rplM^T11E^* in *M. smegmatis* | [9] |
| pVN897 | Hyg^R^, pYUB854 derived, AES for replacement of *Mtb rplM* by *rplM^T11A^* | [9] |
| pVN931 | Amp^R^, pET11c derived, expressing *Mtb* 6H.L13(T11E) from P_T7_ | [9] |
| pVN978 | Kan^R^, pMV361 derived, expressing *M. smegmatis L13(T11E)* from P_hsp60_ | [9] |
| pVN980 | Kan^R^, pMV361 derived, expressing *M. smegmatis renU^DEAD^* from P_hsp60_ | [9] |
| pVN981 | Amp^R^, pET15b derived, expressing *M. smegmatis renU^DEAD^* from P_T7_ | [9] |
| phAE87 | TM4 derived phasmid, conditionally replicative at 30°C | [[8](#_ENREF_8)] |
| phRenU | Hyg^R^, phAE87 derived phasmid, AES for deletion of *renU* in *Mtb* | [9] |
| phL13(T11A) | Hyg^R^, phAE87 derived phasmid, AES for replacement of *rplM* by *rplM^T11A^* in *Mtb* | [9] |

Hyg^R^, hygromycin resistance; Kan^R^, kanamycin resistance; Gen^R^, gentamycin resistance; Amp^R^, ampicillin resistance; AES, allelic exchange substrate. *Mtb*, *Mycobacterium tuberculosis*.

**Reference:**

1. Alland D, Steyn AJ, Weisbrod T, Aldrich K, Jacobs WR, Jr. (2000) Characterization of the *Mycobacterium tuberculosis iniBAC* promoter, a promoter that responds to cell wall biosynthesis inhibition. J Bacteriol 182: 1802-1811.

2. Stover CK, de la Cruz VF, Fuerst TR, Burlein JE, Benson LA, et al. (1991) New use of BCG for recombinant vaccines. Nature 351: 456-460.

3. Nguyen HT, Wolff KA, Cartabuke RH, Ogwang S, Nguyen L (2010) A lipoprotein modulates activity of the MtrAB two-component system to provide intrinsic multidrug resistance, cytokinetic control and cell wall homeostasis in *Mycobacterium*. Mol Microbiol 76: 348-364.

4. Pelicic V, Jackson M, Reyrat JM, Jacobs WR, Jr., Gicquel B, et al. (1997) Efficient allelic exchange and transposon mutagenesis in *Mycobacterium tuberculosis*. Proc Natl Acad Sci U S A 94: 10955-10960.

5. Bardarov S, Bardarov Jr S, Jr., Pavelka Jr MS, Jr., Sambandamurthy V, Larsen M, et al. (2002) Specialized transduction: an efficient method for generating marked and unmarked targeted gene disruptions in *Mycobacterium tuberculosis*, *M. bovis* BCG and *M. smegmatis*. Microbiology 148: 3007-3017.

6. Walburger A, Koul A, Ferrari G, Nguyen L, Prescianotto-Baschong C, et al. (2004) Protein kinase G from pathogenic mycobacteria promotes survival within macrophages. Science 304: 1800-1804.

7. Wolff KA, Nguyen HT, Cartabuke RH, Singh A, Ogwang S, et al. (2009) Protein kinase G is required for intrinsic antibiotic resistance in mycobacteria. Antimicrob Agents Chemother 53: 3515-3519.

8. Bardarov S, Kriakov J, Carriere C, Yu S, Vaamonde C, et al. (1997) Conditionally replicating mycobacteriophages: a system for transposon delivery to *Mycobacterium tuberculosis*. Proc Natl Acad Sci U S A 94: 10961-10966.

9. Constructed in this study.
