# Supplementary material for: A Redox Regulatory System Critical for Mycobacterial Survival in Macrophages and Biofilm Development
Source: PLoS Pathog. 2015 Apr 17;11(4):e1004839. doi: 10.1371/journal.ppat.1004839 (PMC4401782; doi:10.1371/journal.ppat.1004839)
Supplement: S5 Table — (DOCX) [file ppat.1004839.s012.docx]

| **Primer** | **Sequence** |
| --- | --- |
| MS-PknG-del1  MS-PknG-del2  MS-PknG-del3  MS-PknG-del4 | actagtaccaggtggacatcgtcgtcaaga  aagcttgatcagggttctcgggtgaagtca  tctagactggtcgacctggccaacagc  ggtacccaatgggaattgacgggtcgatcc |
| MS-MutT3del1  MS-MutT3del2  MS-MutT3del3  MS-MutT3del4  M_Msm_1  M_Msm_2 | actagtgcaactggatcagcgcctcgtc  aagcttccgttctccgacatcacccagc  tctagatgctcaaccggcagcgctgatc  ggtacccccgtcaggaactcgaagcgc  gatctgcgtctgcatcatcgacga  acctgatccagctgcgtgacaag |
| MS-RplM^T11A^rev  MS-RplM^T11A^fwd  MS-RplM^T11E^rev  MS-RplM^T11E^fwd  MS-RplMrep3  MS-RplMrep4  MS-RplMrep1  MS-RplMrep2  R_Msm_1  R_Msm_2 | taccacgaacgcgtggcgtca  tgacgccacgcgttcgtggta  gtaccacgaacgcgtctcgtca  tgacgagacgcgttcgtggtac  tctagagtgcgtaccggttccctctatg  tcatgagtttcggtcacatcggtcactgg  actagtcagtgcgtcagtagtagggagc  aagcttcgtcctattcatatgccgtttctgg  ccgaaaatctcgcacacaagccaac  cgctctcggtcaccacctcg |
| TB-MutT3del1  TB-MutT3del2  TB-MutT3del3  TB-MutT3del4  M_Mtb_1  M_Mtb_2 | actagtcccagcaattgcaccaacgattcg  aagcttcgtatgcctaacaccatctgtcgg  tctagacggatcagttcgctgctgtaagc  ggtaccggagtggacatcatccagttgcg  gcctctgacagatatgacgacc  ggcaaggctgtatctgtgcacc |
| TB-RplMrep1  TB-RplMrep2  TB-RplMrep3  TB-RplMrep4  TB-RplM^T11A^rev  TB-RplM^T11A^fwd  TB-RplM^T11E^rev  TB-RplM^T11E^fwd  R_Mtb_1  R_Mtb_2 | actagtccatgaagacctcctcagggagg  ctcgagcggaactagcgtgcccaagct  tctagagcgcaacagacccctgtgtggt  ggtaccggctggggtggtttcggtcat  cacgatcgcgtggcgtcaccc  gggtgacgccacgcgatcgtg  cacgatcgcgtctcgtcaccc  gggtgacgagacgcgatcgtg  ccatgaagacctcctcagggag  cgctccaacacgaacgattgtgc |
| TB-PknG-1  TB-PknG-2 | catatggccaaagcgtcagagaccgaa  ctcgagttagaacgtgctggtgggccgga |
| MS-MutT3-1  MS-MutT3-2  MS-MutT3-2-6H | gaattccatatgcgtggcgacggagatggct  aagcttggatccgatcagcgctgccggttgagca ggatccaagctttcaatgatgatgatgatggtggctgctgccgctgccgcgcggcaccaggcgctgccggttgagcaacgg |
| MS-pro-MutT3 | catatgcgatggcctgctcgtagcgct |
| TB-MutT3-1  TB-pro-MutT3  TB-MutT3-2 | gaattccatatgccgacagatggtgttaggcatacg  tctagacaccgacagcgtggtgtacagc  aagcttgcttacagcagcgaactgatccg |
| MS-RplM-1  MS-rplM-1correct  MS-RplM-2 | Gaatcccatatggtgcctacttacacgccgaagg  GAATTCCATAtgcctacttacacgccgaagg  aagcttggatccgtttcggtcacatcggtcactgg |
| TB-RplM-1  TB-RplM-2 | catatgagcgctgtgcccacgtacgc  ggatccggtggtttcggtcattgcgcca |
| TB-RplM^3T^-fwd  TB-RplM^3T^-1  TB-RplM^T11A^-fwd  TB-RplM^T11A^-1  TB-RplM^T12A^-fwd  TB-RplM^T12A^-1  TB-RplM^S14A^-fwd  TB-RplM^S14A^-1 | acgccgcgcgagcgtggta  taccacgctcgcgcggcgtcacccgccttgggcgcgtacgtgggcacagcgctcatatg  acgccacgcgatcgtggta  taccacgatcgcgtggcgtcacccgccttgggcgcgtacgtgggcacagcgctcatatg  acaccgcgcgatcgtggta  taccacgatcgcgcggtgtcacccgccttgggcgcgtacgtgggcacagcgctcatatg  acaccacgcgagcgtggta  taccacgctcgcgtggtgtcacccgccttgggcgcgtacgtgggcacagcgctcatatg |
