# Supplementary material for: A Redox Regulatory System Critical for Mycobacterial Survival in Macrophages and Biofilm Development
Source: PLoS Pathog. 2015 Apr 17;11(4):e1004839. doi: 10.1371/journal.ppat.1004839 (PMC4401782; doi:10.1371/journal.ppat.1004839)
Supplement: S1 Text — (DOCX) [file ppat.1004839.s013.docx]

**Additional Methods**

Quantification of NADH/NAD and FAD

Growing *M. smegmatis* cultures (OD~2) were treated with 1mM H_2_O_2_ for 1 hour to induce oxidative stress before determining levels of these nucleoside diphosphate derivatives. Concentrations of NADH and NAD**^+^** were measured as previously described [[1](#_ENREF_1),[2](#_ENREF_2)], calculated from a standard curve of NADH (Sigma N6660-15VL) and standardized to total protein (mg). FAD levels were determined by a colorimetric method using a kit (K357-100) from Biovision, Inc. (Milpitas, CA). All samples and standards were analyzed in triplicates following the supplier’s instruction. FAD, extracted from cell lysates by perchloric acid, was added to a reaction in which it functions as a cofactor required for an oxidase, which catalyzes the formation of color-generating products. FAD levels was measured through optical absorbance at 570 nm, calculated from a standard curve of FAD, and standardized to total protein (mg).

Intracellular localization of *M. tuberculosis* strains

Macrophage infections and lysosomal trafficking assays were adapted from previously published methods [[3](#_ENREF_3)]. Macrophages were generated by incubating bone marrow monocytes from C57BL/6 mice for 7 days in high-glucose Dulbecco’s Modified Eagle Medium containing 25% L-929 conditioned medium, 1% penicillin, 1% streptomycin, 4.5 g/L glucose, 4 mM L-glutamine, 15% heat-inactivated fetal calf serum, and 0.02 mg/L macrophage-stimulating growth factor (Sigma Aldrich) at 37°C and 10% CO_2_. Macrophages were seeded on MatTek glass bottom 14 mm microwell dishes with coverslip No. 1.5 (MatTek, Ashland, MA) and allowed to adhere for 2 hours (37°C, 10% CO_2_) prior to infection. *Mtb* strains (H37Rv, *Mtb*∆*pknG*, *Mtb*∆*pknG*/*pknG*, *Mtb*∆*renU*, and *Mtb.L13(T11A)*) were grown to saturation and stained with 0.1 mg/ml FLUOS for 15 minutes. Infections were performed at MOI 50:1 (bacillus:macrophage) for 1 hour at 37°C and 10% CO_2_. Infected macrophages were washed 3 times with warm PBS to remove extracellular bacteria, followed by 16-hour chase. After the chase period, lysosomes were labeled for 30 minutes with 1 µM neutral red (Invitrogen) [[4](#_ENREF_4)], and fixed with 4% formaldehyde for 20 minutes. Slides were mounted in ProLong Gold antifade reagent (Invitrogen). Trafficking was analyzed on a Zeiss LSM510 confocal microscope using LSM510 REL3.5 software provided by the manufacturer. Triplicates of 50 events were recorded and analyzed for each condition from triplicate slides.

Induction of PknG Expression and Western Blot

Strains were grown to an OD_600_ of 2, pelleted and three quarters of the medium were removed. Cells were resuspended in the remaining medium and supplemented with 10 mM inducers. Cultures were incubated at 37°C for 30 minutes, unless otherwise stated, with gentle agitation. The induction was stopped by placing the cultures on ice. Preparations of mycobacterial cell extracts were done as previously described [[5](#_ENREF_5)]. Briefly, cells were washed three times in phosphate buffered saline (PBS) plus protease inhibitors (Roche), and disrupted by sonication on ice. Cell lysates were clarified by centrifugation (20,000 r.p.m., 20 min, 4^o^C), treated with SDS sample buffer, and heated at 95 ^o^C for 10 minutes.

SDS-PAGE was performed using the Bio-rad Protean III system. Proteins were separated on 15 % acrylamide gels and transferred onto PVDF membranes. Western Blot was done using standard procedures, using polyclonal anti-PknG [[3](#_ENREF_3)], anti-L13 (Josman LLC, Napa, CA) antibodies, or a monoclonal anti-DivIVA (F126-2) [[5](#_ENREF_5)] antibody. Secondary antibodies coupled to horseradish peroxidase were visualized by chemiluminescence (GE Healthcare Life Sciences).

Kinase Phosphorylation Assays

*In vitro* phosphorylation activity was assayed as previously described [[3](#_ENREF_3)], using purified PknG (0.5 µg) or cell lysates to provide kinase activity. The kinase reaction was carried out for 30 minutes at 37°C in 20 µl buffer (10 mM HEPES, 2 mM DTT, 0.4 mM MnCl_2_, pH 7.5) containing 10 µCi of [γ-^32^P]-ATP (3,000 Ci/nmol, PerkinElmer) and 1.5 µg substrate (purified L13 or RenU). Inhibition of PknG kinase activity was done by preincubation for 15 minutes in the presence of 5 mM AX20017. Reactions were terminated by the addition of SDS-sample buffer and boiled at 95°C for 10 minutes. Proteins were separated on 15% polyacrylamide SDS-PAGE gels and transferred onto PVDF membranes, followed by autoradiography over 6 days. Screens were read on a Storm 820 PhosphoImager and analyzed with ImageQuant software (GE Healthcare Life Sciences).

*In vitro* Nudix Hydrolase Assays

The enzymatic activity and substrate specificity of RenU were investigated using a coupled enzyme colorimetric assay [[6](#_ENREF_6)]. For nucleoside diphosphate derivatives (NDPX), these calf intestinal alkaline phosphatase (CIP) insensitive substrates were first converted to CIP-sensitive products by RenU, followed by the release of phosphate by CIP. For nucleoside triphosphates (NTP), the release of phosphate was catalyzed by inorganic pyrophosphatase (PP_i_ase). In both cases, phosphate was measured by the method of Ames and Dubin [[7](#_ENREF_7)].

The standard incubation mixture contained, in 50 µl, 50 mM Tris pH 8.4, 5 mM MgCl_2_, and 200 nM purified RenU. The mixture also contained 2 mM of a NDPX and 20 U/ml CIP, or 2 mM of a NTP and 20 U/ml PP_i_ase. After 15 minutes at 37^o^C, the reactions were terminated by the addition of 30 µl of 100 mM Na-EDTA. The stopped reactions were kept on ice for 5 minutes, followed by the addition of 700 µl of Ames solution (6 parts 0.42% ammonium molybdate in 1 N H_2_SO_4_ and 1 part 10% succinic acid) [[7](#_ENREF_7)]. The mixtures were then incubated for 20 minutes at 42^o^C. The relative amount of phosphomolybdate was determined by absorbance at 820 nm.

To further characterize the preferred RenU substrates (ADP-Ribose, FAD, and NADH), the 2-step colorimetric assay used for substrate specificity was conducted to determine the Michaelis-Menten parameters V_max_, K_m_, and k_cat_. Optical absorbance readings were collected at one minute intervals using substrate concentrations ranging from 0 to 420 μM. Sodium phosphate was used as a standard to determine the concentration of inorganic phosphate released after hydrolysis and CIP treatment. The initial rates of hydrolysis for different substrate concentrations were fit by nonlinear least squares to the Michaelis-Menten equation (Fig. 3B).

Strain Constructions in *M. smegmatis*

Targeted gene deletion or replacement in *M. smegmatis* was achieved using a recombineering method [[8](#_ENREF_8),[9](#_ENREF_9)]. The 5’-upstream (551 bp, primers MS-PknG-del1 and MS-PknG-del2, *Spe*I/*Hind*III) and 3’-downstream region (528 bp, primers MS-PknG-del3 and MS-PknG-del4, *Xba*I/*Kpn*I) of *M. smegmatis* *pknG* were PCR amplified and cloned directionally to pYUB854 (S4 Table), flanking the built-in hygromycin cassette, to create pVN740. Similarly, the 5’-upstream (606 bp, primers MS-MutT3del1 and MS-MutT3del2, *Spe*I/*Hind*III) and 3’-downstream (500 bp, primers MS-MutT3del3 and MS-MutT3del4, *Xba*I/*Kpn*I) regions of *M. smegmatis* *renU* were cloned to pYUB854 in the same manner, to create pVN755. Next, the linear AESs [5’-DNA-*hyg*Ω -3’-DNA] were removed from pVN740 and pVN755 by *Spe*I/*KpnI* digestion and transformed to a *M. smegmatis* mc^2^155 strain expressing the recombineering system from pVN701B [[8](#_ENREF_8),[9](#_ENREF_9)]. Transformants were selected on 7H10 medium containing hygromycin and kanamycin. Subsequent removal of pVN701B was performed by growing cells at 39ºC in medium containing hygromycin and sucrose. Confirmation of the gene deletions was done by PCRs using primers annealing to chromosomal regions outside of the AESs, followed by sequencing.

For replacement of the wild type *M. smegmatis* *rplM* (encoding L13) by its mutant alleles, the 715 bp-DNA fragments including the 255 bp upstream region of *rplM* (P_rplM_) followed by the mutant genes (*rplM*^T11A^ and *rplM*^T11E^) were cloned by a 2-step PCR method using *M. smegmatis* genomic DNA as template. Primers MS-RplM^T11A^rev and MS-RplM^T11A^fwd (S5 Table) were used together with primers MS-RplMrep3 and MS-RplMrep4 to create P_rplM_-*rplM*^T11A^. Similarly, primers MS-RplM^T11E^rev and MS-RplM^T11E^fwd were used together with primers MS-RplMrep3 and MS-RplMrep4 to create P_rplM_-*rplM*^T11E^. The P_rplM_-*rplM*^T11A^ or P_rplM_-*rplM*^T11E^ was then cloned to pYUB854 using *Xba*I/*Bsp*HI. Next, the 872 bp further upstream of the *rplM* promoter was PCR amplified using primers MS-RplMrep1 and MS-RplMrep2 (S5 Table), and cloned to pYUB854:P_rplM_-*rplM*^T11A^ or pYUB854:P_rplM_-*rplM*^T11E^ at *Spe*I/*Hind*III to create pVN895 or pVN896, respectively. Linear AESs were removed from pVN895 or pVN896 by *Spe*I/*Bsp*H1 digestions and transformed *M. smegmatis* mc^2^155/pVN701B. Mutants were selected and pVN701B was removed as described above. Insertion of Ω*hyg* was confirmed by PCR using primers R_Msm_1 and R_Msm_2 (S5 Table) that anneal to chromosomal sequences outside of the AES, followed by sequencing of the PCR products using primer MS-RplMrep3.

Strain Constructions in *M. tuberculosis*

The *M. tuberculosis* Δ*pknG* (*Mtb*Δ*pknG*) and *M. bovis* BCG Pasteur Δ*pknG* (*BCG*Δ*pknG*) mutants were constructed previously [[3](#_ENREF_3),[9](#_ENREF_9)]. In these strains, the *pknG* genes were replaced by a hygromycin resistance cassette through specialized transduction [[10](#_ENREF_10),[11](#_ENREF_11)].

For deletion of *M. tuberculosis* *renU*, its 502 bp 5’-upstream region was PCR cloned using primers TB-MutT3del1 and TB-MutT3del2, and ligated to pYUB854 [*Spe*I/*Hind*III]. Similarly, the 570 bp 3’-downstream region was cloned using TB-MutT3del3 and TB-MutT3del4 and ligated to pYUB854 [*Xba*I/*Kpn*I]. The obtained plasmid pVN791, which carried the 5’-upstream and 3’-downstream regions of *renU* flanking the of Ω*hyg* cassette, was cloned into the genome of the phage phAE87 at the unique *Pac*I site, and packaged using the GIGAPackIII GOLD system (Stratagene). Recombinant phages, recovered in *M. smegmatis* growing at the permissive temperature (30^o^C), were used to transduce *M. tuberculosis* H37Rv at the non-permissive temperature (37^o^C). Transductants were selected on hygromycin and successful deletion of *renU* was confirmed by PCR using primers M_Mtb_1 + M_Mtb_2 that anneal to chromosomal sequences outside of the AES, followed by sequencing of the PCR products.

For replacement of the wild type *rplM* by its *rplM* ^T11A^ allele, the 716 bp-chromosomal sequence upstream of the putative *rplM* promoter was PCR cloned using primers TB-RplMrep1 and TB-RplMrep2 (S5 Table), and ligated to pUYB854 at *Spe*I/*Xho*I sites to create pYUB-5’T11A. Next, the 676 bp sequence including the putative *rplM* promoter (P_rplM_) followed by the mutant allele *rplM*^T11A^ was generated by a 2-step PCR method using primers TB-RplM^T11A^rev and TB-RplM^T11A^fwd, in combination with primers TB-RplMrep3 and TB-RplMrep4. The cloned P_rplM_- *rplM* ^T11A^ fragment was then subcloned to pYUB-5’T11A [*Xba*I/*Kpn*I] to create plasmid pVN897. Plasmid pVN897 linearized by PacI digestion was ligated to phAE87 genomic DNA digested with the same enzyme, followed by packaging using the GIGAPackIII GOLD system (Stratagene). Recombinant phages carrying the AES recovered in *M. smegmatis* mc^2^155 at the permissive temperature was used to transduce *M. tuberculosis* H37Rv. Transductants were selected on hygromycin and the gene replacement was confirmed by PCR using primers R_Mtb_1 + R_Mtb_2 (S5 Table) that anneal to chromosomal sequences outside of the AES, followed by sequencing of the PCR products using primer TB-RplMrep3.

Plasmid Constructions for *in trans* Expression

Plasmids pVN578 and pVN579, which *in trans* express *M. tuberculosis* and *M. smegmatis* PknG proteins in mycobacteria, respectively, were constructed as previously described [[3](#_ENREF_3)].

Plasmid pVN792 expressing *M. tuberculosis* 6H.PknG was made by PCR amplification of the *pknG* gene from *M. tuberculosis* genome using primers TB-PknG-1 and TB-PknG-2 (S5 Table). The PCR products were cloned to pGEM-T Easy, followed by sequencing. The gene was then subcloned to pET15b [*Nde*I/*Xho*I] to fuse its N-terminus to a 6xHis-tag (6H) encoding sequence on the plasmid.

Plasmid pVN753 expressing *M. smegmatis renU* was made by PCR cloning using primers MS-MutT3-1 + MS-MutT3-2 (S5 Table) and cloned to pMV361 at *Eco*RI/*Hind*III sites, thus coupling its expression to the built-in P_hsp60_ promoter.

A DNA sequence encoding the catalytically-inactive form of RenU, termed RenU^DEAD^ (S2 Table), in which the three glutamate residues of the Nudix box E74, E77, and E78 are mutated to alanines, was chemically synthesized by Biomatik USA, LLC (Wilmington, DE). The DNA fragment was then subcloned to pMV361 (*Eco*RI/*Hind*III) and pET15b (*Nde*I/*Bam*HI) to create pVN980 and pVN981, respectively.

For expression of a RenU.6H in *M. smegmatis* and *E. coli*, *renU* was PCR amplified using primers MS-MutT3-1 + MS-MutT3-2-6H (S5 Table). The DNA was subcloned into pVN747 [*Nde*I/*Hind*III] and pET11c [*Nde*I/*Bam*HI] to create pVN823 and pVN835, respectively. Plasmids pVN823 and pVN835 thus expressed C-terminally 6H-tagged RenU in *M. smegmatis* and *E. coli* from P_SOD_ or P_T7_ promoters, respectively.

For expression of *M. smegmatis* RenU from its native promoter, the gene and its upstream region was PCR cloned using primers MS-pro-MutT3 + MS-MutT3-2-6H (S5 Table) and ligated to pCV125 using at *Nde*I/*Hind*III sites, thus creating pVN866.

Plasmid pVN771, which *in trans* expressed *M. tuberculosis* RenU in mycobacteria, was cloned by PCR using primers TB-MutT3-1 + TB-MutT3-2 (S5 Table) and ligated to pMV361 at *EcoR*I/*Hind*III sites, thus coupling its expression to the P_hsp60_ promoter.

Plasmid pVN840 expressing *M. tuberculosis* RenU from its native promoter was made by cloning the gene and its upstream promoter region using primers TB-pro-MutT3 + TB-MutT3-2 (S5 Table), and ligated to pVN839 at *Nde*I/*Hind*III sites.

Plasmid pVN844 expressing a *M. smegmatis* 6H.L13 in *E. coli* was made by cloning the encoding gene *rplM* from *M. smegmatis* genomic DNA [primers MS-RplM-1 and MS-RplM-2] to pET15b vector at *Nde*I/*Bam*HI sites, thus coupling its expression to the IPTG inducible T_7_ promoter.

Plasmid pVN885 expressing *M. tuberculosis* 6H.L3 in *E. coli* was made by cloning *rplM* from *M. tuberculosis* genomic DNA [primers TB-RplM-1 and TB-RplM-2] into a pET15b vector at *Nde*I/*Bam*HI sites, thus coupling its expression to the IPTG inducible T_7_ promoter.

Mutant alleles of *M. tuberculosis* *rplM* in which codons encoding threonine 11 (T11), threonine 12 (T12) and/or serine 14 (S14) were replaced by those encoding alanine (T11A, T12A, S14A) were generated by a 2-step PCR method. Primers TB-RplM^3T^-fwd, TB-RplM^T11A^-fwd, TB-RplM^T12A^-fwd or TB-RplM^S14A^-fwd (S5 Table) were used together with primer TB-RplM-2 to amplify the C-terminal sequences of *rplM* alleles. Next, the PCR products were annealed with primers TB-RplM^3T^-1, TB-RplM^T11A^-1, TB-RplM^T12A^-1 or TB-RplM^S14A^-1, respectively. Primers TB-RplM-1 and TB-RplM-2 were then added to amplify the entire mutant alleles *rplM*^T11A,T12A,S14A^*, rplM*^T11A^, *rplM*^T12A^ or *rplM*^S14A^. After the introduced mutations were confirmed by sequencing, the mutant alleles were subcloned to pET15b vector at *Nde*I/*Bam*HI sites to create pVN889, pVN890, pVN891 and pVN892, respectively. In these plasmids, expression of L13 mutant proteins was coupled to the IPTG inducible T_7_ promoter.

For overexpression of *M. smegmatis* L13(T11E) in mycobacteria, the coding DNA fragment was re-amplified by PCR from pVN896 using primers MS-RplM-1correct and MS-RplM-2, then subcloned to pMV361 at *EcoR*I/*Hind*III sites to create pVN978.

Expression and Purification of Recombinant Proteins

For expression and purification of *M. tuberculosis* 6H.PknG in *E. coli*, BL21 cells transformed with pVN792 were grown at 37ºC until OD_600_ reached 0.65, followed by induction with 0.1 mM IPTG at 22ºC, 200 r.p.m. for 16 hours, as previously described [[3](#_ENREF_3)]. Cell lysates were prepared in TBS buffer containing protease inhibitor cocktail by sonication (15 cycles of 10 seconds on ice with chilling intervals). After centrifugation, the soluble fraction was diluted 1:1 with wash buffer (50 mM sodium phosphate, 300 mM NaCl, and 45 mM imidazole), and loaded onto a cobalt metal affinity spin column (Pierce), which had been pre-equilibrated with the same buffer. The column was washed 7 times with 2 column volumes of wash buffer and bound protein was eluted 4 times with 1 column volume of elution buffer (50 mM sodium phosphate, 300 mM NaCl, and 250 mM imidazole). Eluted fractions were pooled and stored at -80ºC with 50% glycerol.

For expression and purification of *M. smegmatis* RenU.6H or RenU^DEAD^.6H in *E. coli*, BL21 cells transformed with pVN835 or pVN981 were grown at 37ºC until OD_600_ reached 0.5, followed by induction with 0.5 mM IPTG at 30ºC, 250 r.p.m. for 3 hours. Cell lysates were prepared in lysis buffer (50 mM sodium phosphate, 300 mM NaCl, and 10 mM imidazole) containing protease inhibitor cocktail by sonication (15 cycles of 10 seconds on ice with chilling intervals). After centrifugation, the soluble fraction was loaded onto a nickel metal affinity spin column (Qiagen), which had been pre-equilibrated with the same buffer. The column was washed 6 times with 2 column volumes of wash buffer (50 mM sodium phosphate, 300 mM NaCl, and 20 mM imidazole) and bound protein was eluted 4 times with 1 column volume of elution buffer (50 mM sodium phosphate, 300 mM NaCl, and 500 mM imidazole). Eluted fractions were pooled and stored at -80ºC with 10% glycerol.

For expression and purification of *M. smegmatis* RenU.6H in *M. smegmatis*, 7H9 medium containing hygromycin was inoculated with 5 OD_600_ units per liter of mc^2^155/pVN823 and cultures grown at 37ºC, 240 r.p.m. for 72 hours. Cell pellets were harvested by centrifugation at 4ºC, 4000 r.p.m. for 15 minutes and washed twice with TBS buffer containing a protease inhibitor cocktail (Roche Molecular Biochemicals). After resuspension in 1/10 culture volume of TBS buffer plus protease inhibitors, cells were disrupted by sonication (25 times for 10 seconds on ice with 1 minute cooling intervals), and lysates spun for 20 minutes at 10,000 r.p.m., 4ºC. Supernatant was filtered at 4ºC through 0.22µm filters (Denville) and diluted 1:1 with wash buffer (50 mM sodium phosphate, 300 mM NaCl, and 20 mM imidazole), loaded onto a cobalt metal affinity spin column (Pierce), which had been pre-equilibrated with the same buffer. The column was washed 10 times with 2 column volumes of wash buffer and bound protein was eluted 3 times with 1 column volume of elution buffer (50 mM sodium phosphate, 300 mM NaCl, and 150 mM imidazole). Eluted fractions were pooled and exchanged into 20 mM Tris.HCl buffer (pH 8) using PD-10 desalting columns (GE Healthcare) before loading to a strong anion exchange column (HiTrap Q FF, GE Healthcare) and a 0-1M NaCl gradient. Fractions were analyzed for the presence of RenU.6H by SDS-PAGE, gels stained with Coomassie Brilliant Blue, and fractions containing the purified protein (20 mM Tris.HCl, 100-150 mM NaCl) were pooled and concentrated using 9 kDa molecular weight cut-off spin concentrators (Pierce). Obtained purified fraction was stored at -80ºC with 10% glycerol.

For expression and purification of *M. smegmatis* 6H.L13, *M. tuberculosis* 6H.L13, *M. tuberculosis* 6H.L13(3A), *M. tuberculosis* 6H.L13(T11A), *M. tuberculosis* 6H.L13(T12A) and *M. tuberculosis* 6H.L13(S14A), *E. coli* BL21 cells were transformed with pVN844, pVN885, pVN890, pVN891, and pVN892, respectively, and grown at 37ºC until OD_600_ reached 1. These cultures served as seeds to inoculate (1/100) LB medium supplemented with ampicillin and the inoculated cultures were then grown overnight at 37ºC, 240 r.p.m. Cell lysates were prepared in lysis buffer (50 mM sodium phosphate, 300 mM NaCl, and 10 mM imidazole, pH 9) containing protease inhibitor cocktail by sonication (15 cycles of 10 seconds on ice with chilling intervals). After centrifugation, the soluble fraction was loaded onto a nickel metal affinity spin column (Qiagen) pre-equilibrated with the same buffer. The column was washed 10 times with 2 column volumes of wash buffer (50 mM sodium phosphate, 300 mM NaCl, and 20 mM imidazole, pH 9) and bound protein was eluted 3 times with 1 column volume of elution buffer (50 mM sodium phosphate, 300 mM NaCl, and 500 mM imidazole, pH 9). Eluted fractions were pooled, exchanged into thrombin buffer (20 mM Tris.HCl, pH 8.4, 0.15 M NaCl, 2.5 mM CaCl_2_) using PD-10 desalting columns (GE Healthcare) and 6H-tag was removed by thrombin cleavage (Novagen) at a 5:8 ratio of recombinant protein (mg):thrombin (U) at 4ºC for 20 hours. Cleavage reactions were concentrated using 9 kDa molecular weight cut-off spin concentrators (Pierce) and the cleaved tag and other contaminants were removed by fast protein liquid chromatography (FPLC) using a HiLoad 16/60 Superdex 75 prep grade column (GE Healthcare). Fraction containing purified, untagged protein was concentrated using 9 kDa molecular weight cut-off spin concentrators (Pierce) and stored at -80ºC with 10% glycerol.

For expression and purification of *M. smegmatis* 6H.L13(T11E), *E. coli* BL21 cells were transformed with pVN931 and grown at 37^o^C, 220 r.p.m. until OD_600_ reached 2. This culture served as a seed to inoculate (1/100) LB medium supplemented with ampicillin. The inoculated cultures were treated with 0.5 mM IPTG to induce protein expression at OD_600_ of 0.6 for 3 hours at 37^o^C, 220 r.p.m. Cell lysate was prepared in lysis buffer (50 mM Tris, 500 mM NaCl, 10 mM imidazole, 5% glycerol, pH 8.4) containing protease inhibitor cocktail (Roche) by microfluidization. After centrifugation, the soluble fraction was loaded onto a nickel affinity column (Qiagen) pre-equilibrated with the same buffer. The column was washed 10 times with 2 column volumes of wash buffer (50 mM Tris, 500 mM NaCl, 50 mM imidazole, 5% glycerol, pH 8.4) and bound protein was eluted 10 times with 1 column volume of elution buffer (50 mM Tris, 500 mM NaCl, 10 mM imidazole, 5% glycerol, pH 8.4). The eluted protein was dialyzed into MonoS binding buffer (50 mM Tris, 40 mM NaCl, 5% glycerol, pH 8.4) and loaded onto a MonoS column pre-equilibrated with the same buffer. Protein was eluted with a 20 column volume gradient to an equivalent buffer containing 500 mM NaCl. The eluted protein was pooled and concentrated using 3 kDa molecular weight cut-off spin concentrators (Millipore) for size exclusion chromatography on a Superose 12 column pre-equilibrated in running buffer (50 mM Tris, 300 mM NaCl, 5% glycerol, pH 8.4). Protein was pooled and concentrated as above to 0.61 mg/ml and stored at -80^o^C.

References

1. Burian J, Ramon-Garcia S, Sweet G, Gomez-Velasco A, Av-Gay Y, et al. (2012) The mycobacterial transcriptional regulator whiB7 gene links redox homeostasis and intrinsic antibiotic resistance. The Journal of biological chemistry 287: 299-310.

2. San KY, Bennett GN, Berrios-Rivera SJ, Vadali RV, Yang YT, et al. (2002) Metabolic engineering through cofactor manipulation and its effects on metabolic flux redistribution in Escherichia coli. Metab Eng 4: 182-192.

3. Walburger A, Koul A, Ferrari G, Nguyen L, Prescianotto-Baschong C, et al. (2004) Protein kinase G from pathogenic mycobacteria promotes survival within macrophages. Science 304: 1800-1804.

4. Chazotte B (2011) Labeling lysosomes in live cells with neutral red. Cold Spring Harbor protocols 2011: 207-209.

5. Nguyen L, Scherr N, Gatfield J, Walburger A, Pieters J, et al. (2007) Antigen 84, an effector of pleiomorphism in *Mycobacterium smegmatis*. J Bacteriol 189: 7896-7910.

6. Duong-Ly KC, Gabelli SB, Xu W, Dunn CA, Schoeffield AJ, et al. (2011) The Nudix hydrolase CDP-chase, a CDP-choline pyrophosphatase, is an asymmetric dimer with two distinct enzymatic activities. J Bacteriol 193: 3175-3185.

7. Ames BN, Dubin DT (1960) The role of polyamines in the neutralization of bacteriophage deoxyribonucleic acid. J Biol Chem 235: 769-775.

8. van Kessel JC, Hatfull GF (2007) Recombineering in *Mycobacterium tuberculosis*. Nat Methods 4: 147-152.

9. Wolff KA, Nguyen HT, Cartabuke RH, Singh A, Ogwang S, et al. (2009) Protein kinase G is required for intrinsic antibiotic resistance in mycobacteria. Antimicrob Agents Chemother 53: 3515-3519.

10. Bardarov S, Bardarov Jr S, Jr., Pavelka Jr MS, Jr., Sambandamurthy V, Larsen M, et al. (2002) Specialized transduction: an efficient method for generating marked and unmarked targeted gene disruptions in *Mycobacterium tuberculosis*, *M. bovis* BCG and *M. smegmatis*. Microbiology 148: 3007-3017.

11. Braunstein M, Bardarov SS, Jacobs WR, Jr. (2002) Genetic methods for deciphering virulence determinants of *Mycobacterium tuberculosis*. Methods Enzymol 358: 67-99.
